# Identification and production of novel potential pathogen-specific biomarkers for diagnosis of histoplasmosis

**DOI:** 10.1128/spectrum.00939-23

**Published:** 2023-10-26

**Authors:** Juan David Puerta-Arias, Juan Pablo Isaza Agudelo, Tonny Williams Naranjo Preciado

**Affiliations:** 1 Medical and Experimental Mycology Group, Corporación para Investigaciones Biológicas (CIB-UdeA-UPB-UDES), Medellín, Colombia; 2 School of Health Sciences, Universidad Pontificia Bolivariana, Medellín, Colombia; 3 Universidad de Santander (UDES), Facultad de Ciencias Médicas y de la Salud, Bucaramanga, Colombia; Geisel School of Medicine at Dartmouth, Lebanon, New Hampshire, USA

**Keywords:** histoplasmosis, biomarkers, fungal antigens

## Abstract

Immunological assays based on the detection of circulating fungal biomarkers are helpful in clinical practice for diagnosing invasive fungal infections. Some of these targeting antigenic components are common to several different fungi. Histoplasmosis is a mycosis caused by the dimorphic fungus *Histoplasma capsulatum*, which in recent years has gained significant relevance due to the increase in the population susceptible to developing severe clinical forms, including those living with HIV/AIDS. Immunological tests that detect cell wall polysaccharide antigens are among the most used laboratory techniques for diagnosing this mycosis. However, none have shown adequate performance, and cross-reactivity with other fungal pathogens may be observed. Considering that there is a real need to improve the sensitivity and specificity of current diagnostic methods, we explored a novel strategy for the identification of *H. capsulatum-*specific antigens (*Hc_Ags*) that could be detected in clinical samples during infection based on a computational analysis model that included the application of bioinformatics tools and the analysis of experimental data from transcriptomics and proteomics. The *Hc_Ags* identified were expressed and purified by eukaryotic and prokaryotic systems. First, the *Hc_Ags* were used in an in-house immunization model in mice (BALB/c) to obtain *Hc_Ag*-specific polyclonal antibodies (*Hc_Ag_PAb*). Then, the presence of these antigens in *H. capsulatum*-yeast culture extracts and the specificity of *Hc_Ag_PAb* against culture extracts of *Candida albicans*, *Aspergillus fumigatus*, *Cryptococcus neoformans*, *Fusarium* spp., and *Paracoccidioides brasiliensis* were confirmed. Finally, we demonstrated the immunoreactivity of these *Hc_Ag*-specific polyclonal antibodies with urine samples from patients previously diagnosed with histoplasmosis.

**IMPORTANCE:**

Histoplasmosis is considered one of the most important mycoses due to the increasing number of individuals susceptible to develop severe clinical forms, particularly those with HIV/AIDS or receiving immunosuppressive biological therapies, the high mortality rates reported when antifungal treatment is not initiated in a timely manner, and the limitations of conventional diagnostic methods. In this context, there is a clear need to improve the capacity of diagnostic tools to specifically detect the fungal pathogen, regardless of the patient’s clinical condition or the presence of other co-infections. The proposed novel pathogen-specific biomarkers have the potential to be used in immunodiagnostic platforms and antifungal treatment monitoring in histoplasmosis. In addition, the bioinformatics strategy used in this study could be applied to identify potential diagnostic biomarkers in other models of fungal infection of public health importance.

## INTRODUCTION

Currently, immunological techniques based on the antigen-antibody reaction are widely used in routine laboratories to diagnose multiple infectious diseases. The capacity of these methods to detect specific circulating antimicrobial antigens directly from clinical samples can lead to rapid diagnosis and effective treatment for patients (1).

In the case of fungal infections, many immunodiagnostic tests have been developed based on the detection of cell wall components, mainly polysaccharides such as 1,3-β-D-glucan and galactomannan, which are present in spores, hyphal filaments, or yeast-like cells (2). However, since several of these surface antigens are ubiquitous in a broad spectrum of fungal species, false-positive results are typical, causing difficulties in differentiating the correct pathogenic fungus (3).

This study focuses on the thermally dimorphic fungus *Histoplasma capsulatum*, the causal agent of histoplasmosis. This mycosis, highly endemic in American countries, affects people with impaired immunity, especially those living with HIV/AIDS (4 – 6). Currently, several conventional laboratory methods have been developed for diagnosing this fungal infection, including culture, histopathology, and immunological and molecular tests. However, the performance of these methods often depends on the clinical form (acute or chronic infection), the patient’s immune status, or the laboratory’s mycological expertise (7 – 9). Regarding the immunological tests developed for the diagnosis of histoplasmosis, few laboratories offer detection services for *Histoplasma* circulating antigen, and some are not available for Latin American countries (10 – 15). Additionally, these tests, including the most widely used, such as the *Histoplasma-*polysaccharide antigen enzyme-linked immunoassay (EIA) developed by MiraVista Diagnostics (Indianapolis, USA) (16) and the commercial EIA test designed by IMMY company (Immuno-Mycologics, IMMY, Norman, OK, USA) to detect *Histoplasma-galactomannan* antigen (11), have reported a variable analytical sensibility (40%–86%) and cross-reactivity in samples from patients with other fungal infections, including blastomycosis, coccidioidomycosis, paracoccidioidomycosis, penicilliosis, cryptococcosis, aspergillosis, sporotrichosis, and emergomycosis (16 – 22). Moreover, the MVista EIA test, which uses polyclonal antibodies (PAbs) to detect *H. capsulatum* polysaccharide antigens (HPA), is exclusively available in the United States of America, while IMMY EIA test is a commercial test that uses monoclonal antibodies (MAbs) and has been validated in some Latin American laboratories, mainly with urine samples. However, it is not recommended for other biological specimens due to the variable sensitivity demonstrated (10, 23, 24).

Considering that treatments may differ between certain fungi or not be effective, it is imperative to design new diagnostic tools with greater specificity and suitable for conventional laboratories in endemic countries (25, 26). Furthermore, the main challenge is to identify which molecules produced by the fungus during infection could be used as potential antigen targets for immunoassays. However, to our knowledge, only a few studies have been published focused on identifying new potential biomarkers that may be useful not only for immunodiagnosis but also for monitoring the effectiveness of treatment in fungal infections such as histoplasmosis.

Here, we applied a bioinformatics strategy that integrates the use of computational tools such as OrthoMCL (27), BLASTp (Basic Local Alignment Search Tool protein) (28, 29), TargetP (30), and SignalP (31), applied to a local collection of proteome database obtained manually from GenBank-NCBI, and the analysis of previously published biological and experimental data sets [omics data (genomics, transcriptomics, and proteomics)] (32) applied to the search, selection, and production of potential biomarkers for the immunodiagnostic of histoplasmosis.

The *Histoplasma* antigens described in this study could serve as proof of principle for new diagnostic tools based on immunological methods. Furthermore, our results showed that this strategy is a means for identifying specific biomarkers for immunoassays and could be applied to other infection models.

## RESULTS

### Analysis of orthologous proteins

**Fig 1:**
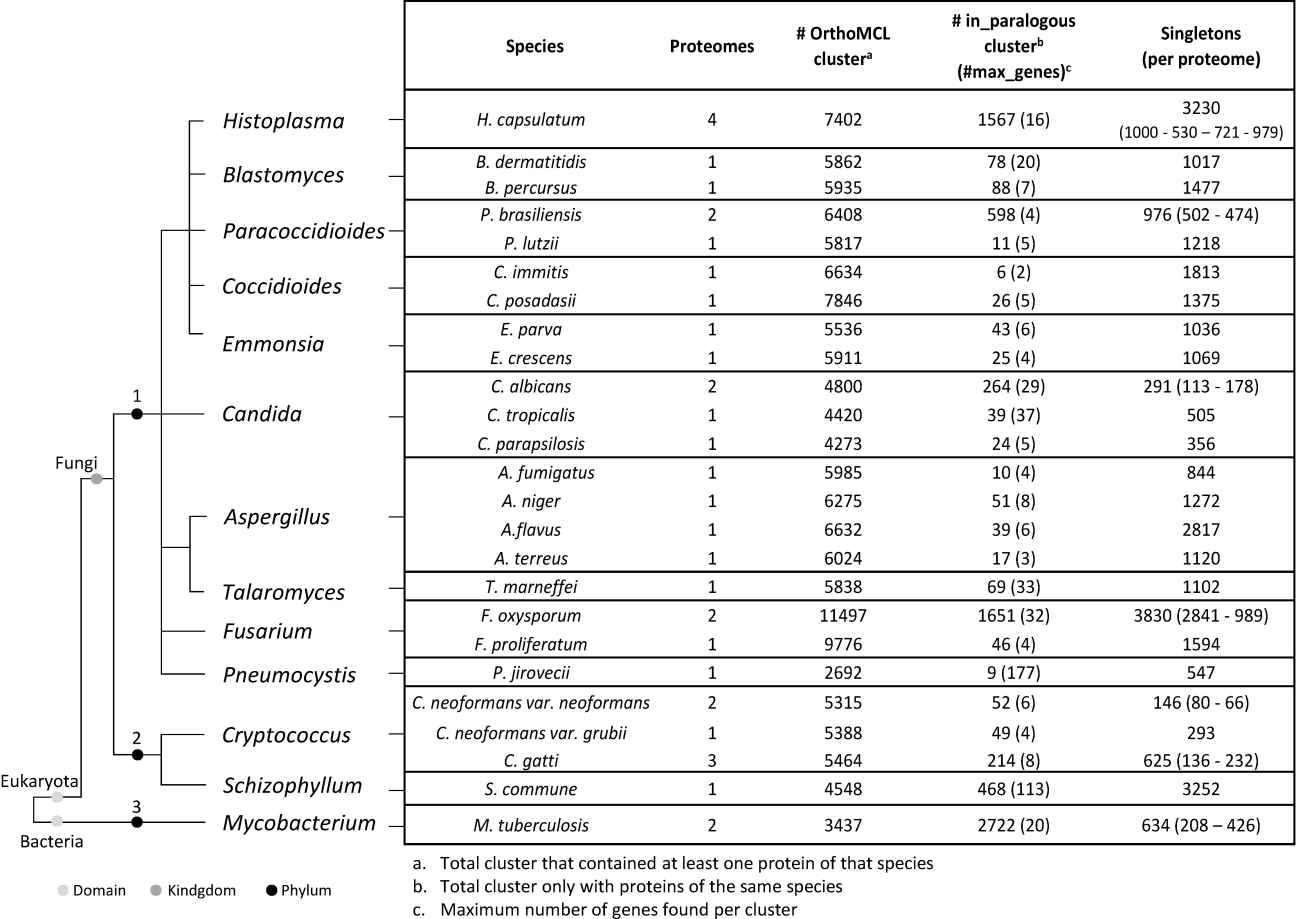
Descriptive analysis of OrthoMCL clusters. A non-statistical cladogram is collapsed, showing the relationships and taxonomic classification among the fungal species included in the analysis. The number indicates the phylum; 1: *Ascomycota*, 2: *Basidiomycota*, and 3: *Actinobacteria*. On the right-hide side, the table compares the number of gene families identified by OrthoMCL for each species.

A comparative analysis of 343,723 proteins obtained from 33 proteomes of fungi species and 2 proteomes of *Mycobacterium tuberculosis* was performed using the OrthoMCL algorithm (Table S1). Of the 25,770 OrthoMCL protein clusters obtained, 1,851 (7%) contained at least one protein from each species included in the analysis. Not surprisingly, these proteins are involved in central biological processes, including cell cycle regulation, transport, cytoskeleton organization, or as surface antigens (data not shown). Further exploration of the data sets showed that most clusters contained between two and nine proteins (*n*: 19,618 clusters, 76%), and only a group of five clusters included more than 1,000 predicted orthologs genes (Table 1). For each fungal species, the clusters with at least one protein, the clusters with in-paralogs proteins (genes present in a particular fungal species that are related to each other through a gene duplication event, without orthologs genes found in different species), and the total number of singleton proteins (genes for which no orthologous or paralogous relationship was found) are presented in Fig. 1. Interestingly, *H. capsulatum* presented a group of 1,567 protein clusters containing a total of 3,572 in-paralogs proteins and 3,230 singletons proteins, which were determined as specific to this fungus by OrthoMCL. A total of 6,802 *Hc-proteins* were selected for the subsequent analysis.

**TABLE 1:** Total number of genes per cluster

Number of genes	Number of clusters	%
2,428	1	0.004
>1,000	4	0.016
501–1,000	18	0.07
300–500	18	0.07
200–299	36	0.14
100–199	134	0.52
10–99	5,941	23
2–9	19,618	76

### Identification of potential biomarkers

**Fig 2:**
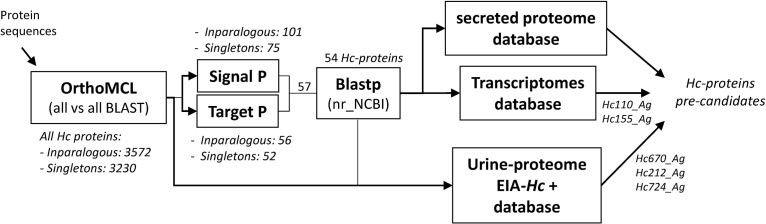
Flowchart of the analysis pipeline. Overview of the workflow of bioinformatic analysis system used to identify *H. capsulatum*-specific gene as a potential biomarker diagnosis of histoplasmosis. For each analysis step, the information obtained is shown. nr_NCBI indicates the BLAST analysis against the nr database compiled by NCBI. Secreted proteome database, transcriptomes database, and urine-proteome EIA-Hc + database indicate the comparative analysis against the database previously published by Holbrook et al. (33), Edwards et al. (34), and Crockett et al. (35), respectively.

We designed a strategy to find those proteins specific to *H. capsulatum* that can serve as potential biomarkers for diagnosis. The overall analysis pipeline is illustrated in Fig. 2.

Following OrthoMCL analysis, the next step was to use TargetP and SignalP to detect *H. capsulatum* proteins that contain a signal peptide (SP) in the N-terminal region, which is responsible for exporting proteins to extracellular environment. Thus, if a gene had this signal, it was considered a secretory protein. SignalP output revealed 176 genes (101 in-paralogs and 75 singletons) predicted to be classically secreted proteins. In contrast, TargetP predicted 108 proteins that contained secretory pathway signal peptides (56 in-paralogs and 52 singletons). The differences between the two programs could be that SignalP predicts the presence of N-terminal (and cleavable) signal peptides, while TargetP includes the prediction of transmembrane segments associated with other subcellular localizations (cytoplasmic space or cellular membrane) (36). Only proteins predicted by both programs were selected to confirm the analysis further. A total of 57 *Hc-proteins* were identified as putative secreted proteins (34 in-paralogs and 23 singletons). (Fig. 2). Then, BLASTp against NCBI non-redundant (nr) database was used to rule out proteins that shared similarities with proteins from other species that were not included in the initial OrthoMCL analysis. At last, 54 proteins (31 in-paralogs and 23 singletons) were considered specific to *H. capsulatum* (Fig. 2).

As a final step, we analyzed previously published experimental data sets and combined them with our orthological analysis to identify potential biomarkers for diagnosing histoplasmosis (Fig. 2). At first, the previously published *H. capsulatum* extracellular proteome database was used (33). Unfortunately, all proteins described as the core set of extracellular proteins produced by this fungus were found in clusters with similar proteins from other fungi and were initially discarded within the analysis.

We also used a transcriptome database with the phase-specific gene profiles (yeast or mycelium) of *H. capsulatum* previously published by Edwards et al. (34). Interestingly, two genes encoding proteins previously defined in our study as *H. capsulatum*-specific were identified within a subset of differentially expressed genes. One of them showed significantly enriched expression (50-fold) in the yeast phase compared to the mycelia phase (*Hc110_Ag*), and the other was a yeast-phase-specific gene (*Hc155_Ag*). Both genes were considered potential candidates for biomarker diagnosis (Table 2).

**TABLE 2:** Description of potential diagnostic biomarkers for histoplasmosis

Hc_Ags^*a*^	ID_NCBI	kDa^*b*^	OrthoMCL cluster	Analysis databases
Hc110_Ag	gb|EEH06109.1	12	*Histoplasma* in-paralogous gb|EGC48864 gb|EER37578	FPKM^*c*^ 255:0
Hc115_Ag	gb|EEH03588.1	17	Singletons	FPKM^*c*^ 232:4
Hc670_Ag	gb|EDN08842.1	75	Singletons	Urine-Hc-peptides^*d*^ LLFVGSNSAPGR TLPDDHILQEAK
Hc724_Ag	gb|EDN06621.1	81	Singletons	Urine peptide^*d*^ KLSTLVGALATRN
Hc212_Ag	gb|EDN09437	23	*Histoplasma* in-paralogous Hc04|EDN09437.1 Hc01|EEH04145.1 Hc02|EGC48357.1 Hc01|EEH03007.1 Hc03|EER42161.1 Hc02|EGC43734.1 Hc03|EER40420.1 *Paracoccidioides* homologs gb|XP_015700594.1 gb|EEH44665.2 gb|KGY15563.1 *Blastomyces* homologs gb|EGE86184.2 gb|OJD22518.1 *Emmonsia* homologs gb|KLJ06008.1 gb|KKZ65985.1 *Cryptococcus* homologs gb|ADV24578.1 gb|KIR57842.1	Urine-Hc-peptides^*d*^ MFYFDSEFVGPPR LLWGGAQQER

^*a*^ Name defined by authors.

^*b*^ Molecular weight based on the amino acid sequence.

^*c*^ Gene expression calculated as fragments per kilobase of exon per million fragments mapped (FPKM). Value as yeast:mycelia ratio (32).

^*d*^ *Histoplasma*-specific peptides identified by Crockett et al. (36).

Furthermore, a urine-peptides database from immunoassay-positive patients with disseminated histoplasmosis previously published by Crockett et al. was used (35). Considering that this report was able to identify several *Histoplasma* proteins, which are not necessarily secreted but represent the urine-proteome associated with *Histoplasma*-antigenuria, a comparative analysis was performed with the total all-*Hc-proteins* identified by OrthoMCL plus an nr_BLASTp (NCBI database) analysis. The results showed that three urine peptides were closely matched with two *Hc-*specific proteins according to our analysis (*Hc670_Ag* and *Hc724_Ag*), and both proteins were considered diagnostic biomarkers of histoplasmosis and selected for the subsequent experimental process (Table 2).

Unsurprisingly, most of the peptides published in that study corresponded to proteins that shared homology with proteins from other fungi (Table S2). Due to this, all protein clusters obtained by OrthoMCL paired with any urine peptides of *H. capsulatum* were further analyzed. Finally, we found that one of these clusters, related to two urine peptides, contained 16 homologs proteins (according to OrthoMCL) from the fungal genera; *Paracoccidioides* (3), *Blastomyces* (2), *Emmonsia* (2), *Cryptococcus* (2), and seven in-paralogs *H. capsulatum*, designed at *Hc212_Ag*. A subsequent BLASTp analysis revealed that the percent match and query coverage were <40% between those proteins. Considering that an antigenic epitope can be defined as a short sequence of 10 to 16 amino acids (linear and/or conformational), the low sequence similarity observed between the proteins of the cluster, and the size of the identified protein (212 aa), this *Hc-protein* (*Hc212_Ag*) was considered a potential diagnostic biomarker (Table 2).

### Protein expression and immunoreactivity

**Fig 3:**
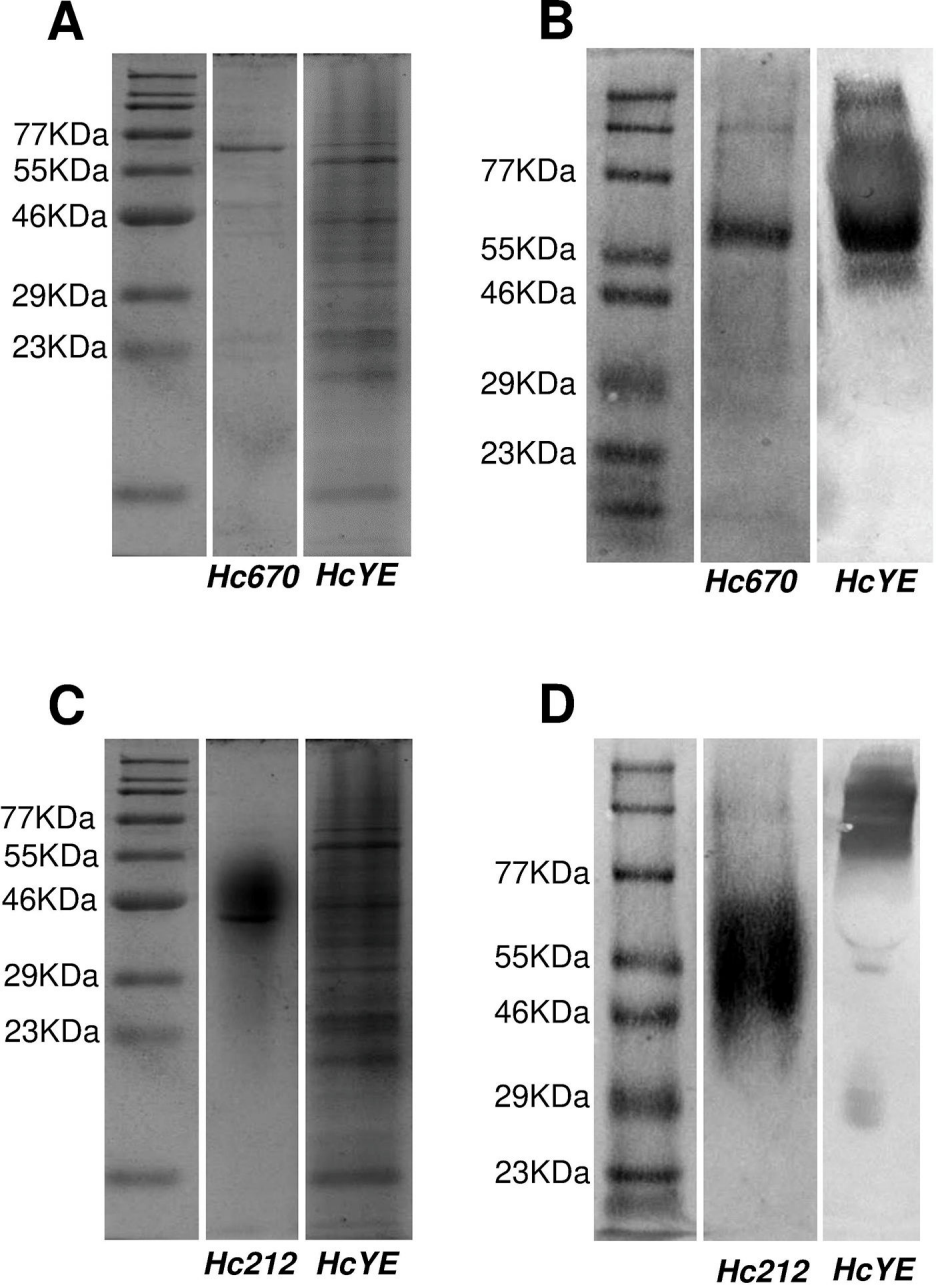
Protein expression and reactivity of PAbs anti-*Hc670_Ag* and anti-*Hc212_Ag*. SDS-PAGE (A and C) and western blotting (B and D) of *Hc670_Ag* (A and B) or *Hc212_Ag* (C and D) purified proteins, and culture extract collected from *H. capsulatum* yeast (*HcYE*) running at 12% with protein size standards molecular weight markers. Same *HcYE* and pre-stained protein ladder were used for analysis and running of SDS-PAGE. Specific reactivity of PAb of (B) anti-*Hc670_Ag* or (D) anti-*Hc212_Ag* obtained from mice immunized was evaluated.

Initially, the construct of the expression vector (pET-100D/TOPO plasmid) for each candidate was obtained by GeneArt (Gen Synthesis Services, USA). All candidates for diagnostic biomarkers, except for *Hc212_Ag*, were expressed by a prokaryotic recombinant protein expression system, induced with IPTG in *Escherichia coli* BL21 (strain DE3) and purified by affinity column chromatography (as described in Materials and Methods). The *Hc212_Ag* was obtained by external service of recombinant protein expression in eukaryotic cells (GenScript, Biotech Corporation, USA) since it presented post-translational glycosylation-type modifications. The expression of purified recombinant proteins, *Hc670_Ag* and *Hc212_Ag*, was confirmed by SDS-PAGE, and the molecular weights were found to be around 75 kDa and 40 kDa, respectively (Fig. 3A and C). *Hc110_Ag*, *Hc155_Ag*, and *Hc724_Ag* were not possible to express and purify at the time of this publication, so they will be used in other future studies.

**Fig 4:**
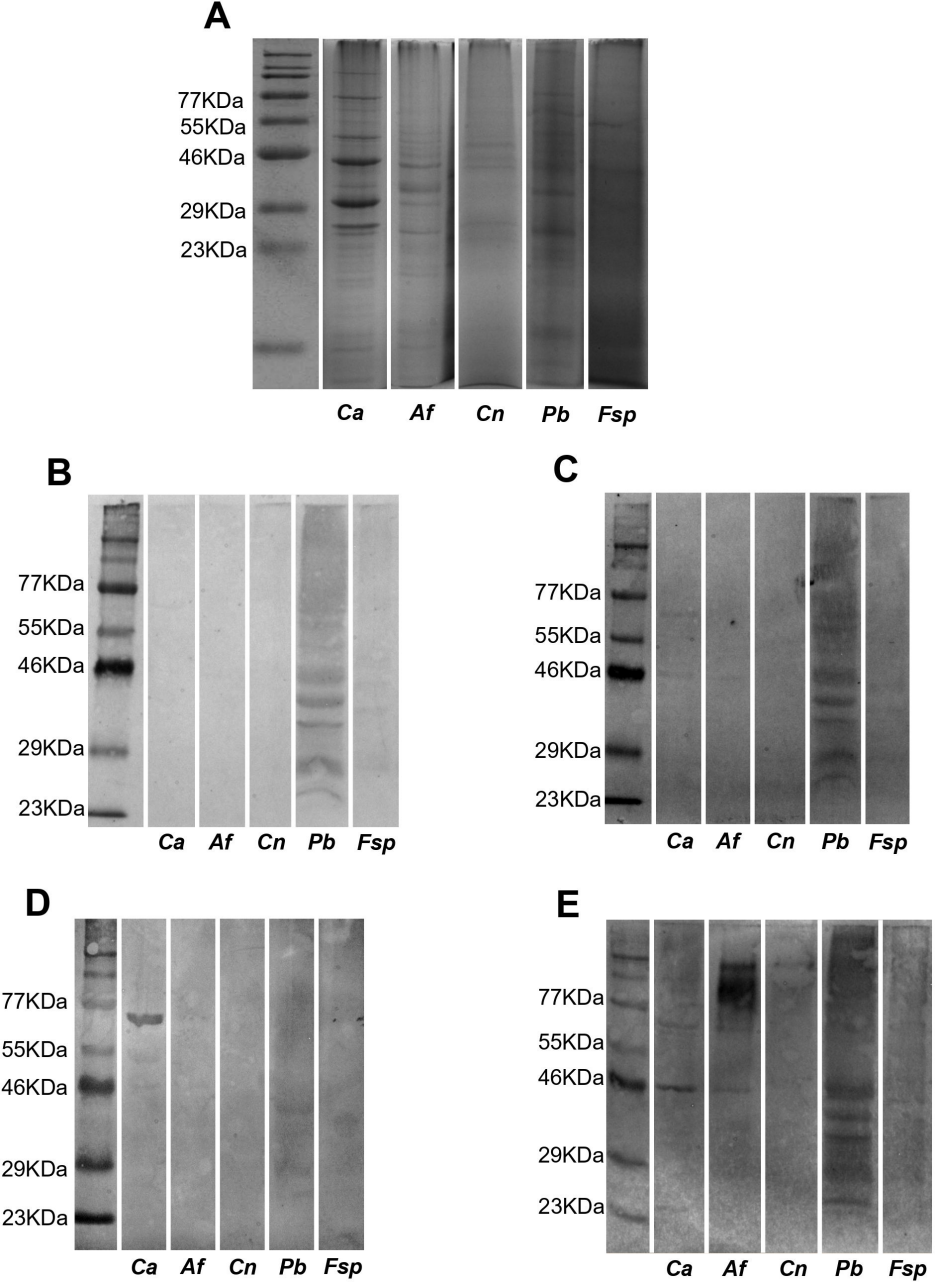
Immunoreactivity of PAbs anti-*Hc670_Ag* and anti-*Hc212_Ag* to culture extracts of *C. albicans*, *A. fumigatus*, *P. brasiliensis*, *C. neoformans*, and *Fusarium* spp. SDS-PAGE (A) and western blotting (B–E) of culture extracts of *C. albicans* (*Ca*), *A. fumigatus* (*Af*), *Cryptococcus neoformans* (*Cn*), *Paracoccidioides brasiliensis* (*Pb*), and *Fusarium* spp. (*Fsp*) running at 12% with molecular weight markers. Same *HcYE* and pre-stained protein ladder were used for analysis and running of SDS-PAGE. Specific reactivity of PAb of anti-*Hc670_Ag* (B), anti-*Hc212_Ag* (C), or commercial monoclonal antibody anti-*C*. *albicans* (D) and serum anti-*Aspergillus* (E) were evaluated.

The SDS-PAGE of purified proteins and culture extracts of *H. capsulatum* (yeast), *Candida albicans* (yeast), *Aspergillus fumigatus* (mycelium), *Cryptococcus neoformans* (yeast), *Paracoccidioides brasiliensis* (yeast), and *Fusarium* spp. (mycelium) were immunoblotted with immunized mice’s sera and the reactivity of the PAbs anti-*Hc670_Ag* and anti-*Hc212_Ag* was observed only with *H. capsulatum* proteins (*Hc212_Ag* and *Hc670_Ag*) and *H. capsulatum* yeast extracts (Fig. 3B and D) but not with the other fungal extracts (Fig. 4B and C), except for *P. brasiliensis*, which showed multiple reactivity bands with both PAbs, anti-*Hc670_Ag* and anti-*Hc212_Ag*. As a control, commercial monoclonal antibody anti-*C*. *albicans* (Fig. 4D) and serum anti-*Aspergillus* from patients with confirmed aspergillosis invasive (Fig. 4E) were used against fungal extracts.

**Fig 5:**
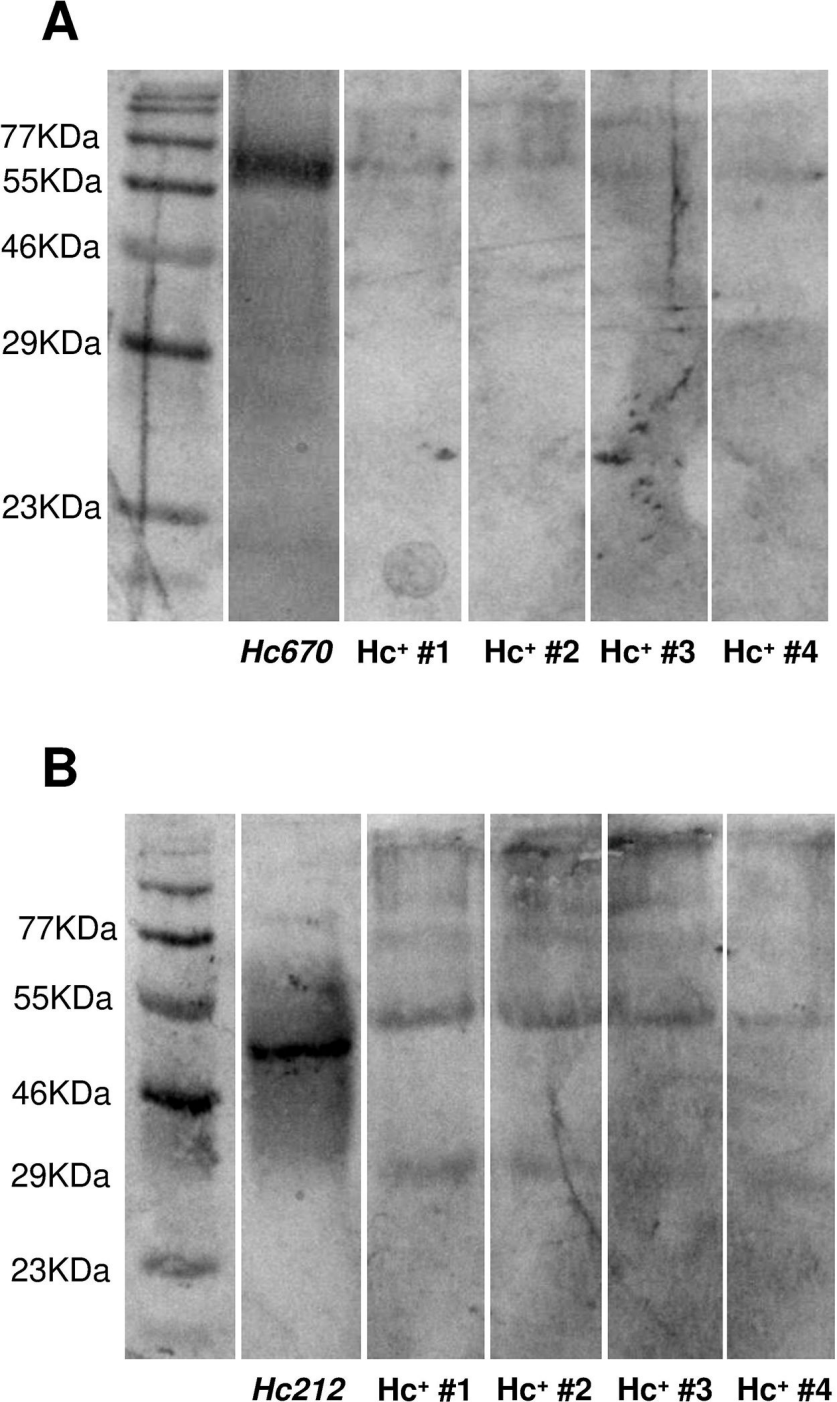
Immunoreactivity of PAbs anti-*Hc670_Ag* and anti-*Hc212_Ag* to samples from patients with confirmed histoplasmosis. Immunoblots of individual urine samples from patients with a confirmed diagnosis of histoplasmosis (Hc^+^ #1 to #4) demonstrated reactivity to PAbs *anti-Hc670_Ag* (A) and *anti-Hc212_Ag* (B). Purified proteins were used as a control.

Finally, preliminary experiments were performed with anonymized urine samples from patients with confirmed disseminated histoplasmosis by IMMY ALPHA EIA test. It was observed that both anti-*Hc670_Ag* and anti-*Hc212_Ag* were immunoreactive with the samples (Fig. 5A and B), indicating that both specific *H. capsulatum* proteins have potential use as diagnostic biomarkers for human histoplasmosis.

## DISCUSSION

In fungal infections, the concept of circulating antigens in infected host serum as potential biomarkers of disease has been reported for many years (37 – 40). Currently, immunoassay tests involving fungal antigen detection are widely used in clinical practice to diagnose and monitor antifungal treatment.

In the case of histoplasmosis, the detection of circulating antigen has been described as the best option for diagnosis, mainly in immunocompromised patients with disseminated histoplasmosis (9, 41). There are two major EIA tests based on the detection of low molecular weight HPA present in the urine and serum of patients, which have been validated in two laboratories: Mvista Histoplasma Quantitative EIA test (Miravista Diagnostics, Indianapolis, USA) and IMMY ALPHA Histoplasma Antigen EIA test (Immuno-Mycologics, Inv., Norman, OK) (42). Nevertheless, several authors have reported discrepant results with samples analyzed with tests and cross-reactions with other fungal infections (11, 14, 43 – 45). Recently, one of these companies developed a new lateral flow assay for detecting HPA in urine samples (46, 47) that may allow a point-of-care diagnosis of histoplasmosis. However, cross-reactivity is still one of the significant challenges.

Likewise, it has been described that other antigens of *H. capsulatum* commonly used in serodiagnosis, such as M and H antigens, exhibit cross-reactivity with other dimorphic fungi (48, 49). Considering that currently no specific and clearly defined biomarkers of *H. capsulatum* are known, we used several analytical approaches to identify potential biomarkers for the diagnosis of histoplasmosis by applying different bioinformatics tools, including computational platforms for prediction of orthologs and secretory proteins and comparative analysis with multiple experimental data sets that provide a comprehensive proteomic-scale analysis.

Indeed, the recent advances in next-generation sequencing technologies, the increasing genome and protein entries into NCBI (National Center of Biotechnology Information), and the development of new bioinformatics algorithms have facilitated comparative genomics and proteomics studies. The OrthoMCL is an algorithm widely used for identifying orthologs of proteins between multiple species based on sequence similarity (all vs all BLASTp), which also allows recognition of those specific proteins for each species (27, 50 – 52). Our study used a predicted proteome of four *H*. *capsulatum* strains (*G186A*, *H88*, *H143*, and *Nam1*). These proteomes exhibited differences in genome length (Mbp) and the number of annotated proteins that could be related to a non-identical workflow of sequencing and assembly defined by each NCBI-Bioproject per strain (53).

By OrthoMCL analysis, the *H. capsulatum* proteomes were compared with other proteomes of clinically significant or phylogenetically related fungal pathogens that also infect immunocompromised patients and cause nonspecific and indistinguishable symptoms (8, 15, 54). Additionally, despite the noticeable and recognized difference between fungi and bacteria, the *Mycobacterium tuberculosis* proteome was included in the analysis, considering the high incidence of histoplasmosis with pulmonary tuberculosis cases reported among people living with HIV/AIDS and the similarity of the clinical and radiological presentations between both (55, 56). The results obtained showed that 1,851 of the 25,770 protein groups formed by OrthoMCL contained proteins from each fungus species with high genetic similarities and were considered homologs proteins, which could be a consequence of common ancestry, horizontal transfer in a shared habitat, or a mixture of both (57, 58). Additionally, the present analysis showed that for each fungus species, a group of unique proteins called “singletons” could be used in future studies focused on identifying new diagnostic biomarkers.

Particularly, *H. capsulatum* showed a total of 3,230 singleton proteins and multiple in-paralogs protein clusters with high sequence similarities (1,567 clusters for a total of 3,572 proteins). However, each protein in these paralogs clusters was evaluated individually. Alternately, a BLASTp analysis using NCBI-compiled nr databases containing non-redundant RefSeq protein records from GenBank and other protein data bank archives (*PDB*, *Swiss-Prot*, *UniProt*, etc.) was considered in our analysis, which improved the precision of the study by allowing the identification of particular *H. capsulatum* proteins (59, 60).

Our strategy also included the prediction of putative secreted proteins that contained the signal sequence and were identified as targeting the classical secretory pathway. All *Hc-proteins* were analyzed with the amino-acid sequence-based predictors SignalP and TargetP. Thus, only those proteins that had the following characteristics: (i) an N-terminal SP; (ii) no transmembrane domains; and (iii) no predicted localization signal to other intracellular organelles, were considered “secreted *Hc-proteins.*” Further analysis of non-classical pathways was not considered since few proteins secreted by this pathway have been described in fungi and the current bioinformatics programs focused on this approach, such as SecretomeP 2.0, are not fully ported and only work for mammalian and Gram-negative proteins (61, 62). Overall, this prediction approach would allow us to target possible circulating antigens the fungus releases during an infection.

Even with the robustness and extensive use of computational biology, it is essential to validate the scientific finding through empirically based knowledge or experimental methods that enhance the confidence and accuracy of predictive computational models (63, 64). Accordingly, we used a previously published, unique, and representative data set of peptides obtained directly from urine samples of patients with disseminated histoplasmosis (35, 65). In this study, the authors reported that some peptides contained sequences homologous to conserved hypotheticals *H. capsulatum* proteins. However, the specificity of these peptide-related proteins was not fully verified. Interestingly, through this data set, we identified three proteins, two of them considered by OrthoMCL as singletons proteins of *H. capsulatum* (*Hc670_Ag* and *Hc724_Ag*), that would have experimental evidence as circulating antigens in the urine (and presumably in the serum) of infected patients.

Both *Hc670_Ag* and *Hc724_Ag* are classified as predicted proteins with unknown biological function, molecular or biochemical structure, or recognized orthologs or homologs (based on sequence similarity). Several reports have described that approximately 80% of *H. capsulatum* yeast-phase-regulated genes encode hypothetical and uncharacterized unknown proteins, highlighting how little is currently understood about the biology of *H. capsulatum* and the need for further studies focused on characterizing at the molecular level and gaining a comprehensive view of genes that can serve as potential biomarkers for diagnosis (33, 34, 66, 67). Coincidentally, some authors have reported the production of murine monoclonal antibodies with potential use in epidemiology and serodiagnosis that recognized an *H. capsulatum* antigen with an apparent molecular mass of 70–75 kDa, like *Hc670_Ag*, but exhibited weak reactivity to antigens derived from *Sporothrix*, *Paracoccidioides*, *or Blastomyces* (68 – 70). However, these studies utilized a whole yeast cell extract that could contain antigens shared with other fungal pathogens, contrary to the use of individual *H. capsulatum*-specific antigens as proposed in this study.

Regarding the other identified protein, *Hc212_Ag*, it is a predicted glycosylated protein that was matched with a secreted and yeast-phase-specific protein with unknown functions previously designated as *Cfp-4* (Culture filtrate protein) (33), and linked to an ortholog group of proteins from *H. capsulatum* and other fungi. However, some reports have described that OrthoMCL can cluster genes with high-scoring (bit-score) alignments that are not homologs or share functional similarities (59, 71, 72). Therefore, by analyzing all protein clusters paired with any of the *H. capsulatum* urine peptides, we found that, particularly, a protein cluster with seven in-paralogs proteins of *H. capsulatum* (homologs to *Cfp-4*) and nine putatively homologs proteins from other fungi [*Paracoccidioides* (3), *Blastomyces* (2), *Emmonsia* (2), and *Cryptococcus* (2)], unexpectedly had a low sequence similarity. The above, added to the fact that *Cfp-4* was described as one of the main extracellular factors produced by *H. capsulatum*, although it does no apparent role in the virulence or pathogenesis of infection (33, 73), could be considered as an *H. capsulatum*-specific yeast-phase exoantigen. More recently, Gallo et al. reported the design of primer sets for two genes, *PPK* (predicted protein kinase) and *Cfp-4*, to conventional and real-time PCR assays for the identification of *H. capsulatum* isolates using purified DNA (74). Similarly, Rubio-Carrasquilla et al. observed that there are no proteins similar to *Cfp-4* in any other organism (BLAST search) and identified it as an *H. capsulatum*-specific immunogenic protein (75). However, to our knowledge, there is no investigation evaluating this protein’s potential utility as a diagnostic histoplasmosis biomarker.

Under this same approach, we identified two other proteins (*Hc110_Ag* and *Hc115_Ag*) predicted as putative secreted proteins of *H. capsulatum* and differentially upregulated in the yeast phase (related to active infection) that could have great potential as diagnostic biomarkers. Until now, non-identical or similar proteins have been reported previously. However, as of this publication’s date, it was impossible to fine-tune the protein synthesis and purification processes and verify their presence in yeast culture extracts. Thus, these will be analyzed in further studies.

On the other hand, other strategies focused on identifying extracellular antigens released during infection have been published with *Candida* and *Aspergillus*. These strategies were based on producing hybridoma cell lines and MAbs from specific structural and metabolic components, such as germ-tube-specific antigens, hyphal cell wall antigens, or galactomannan-like antigens (3, 76 – 78). However, lyophilized mycelium or ethanol-precipitated exoantigens from mycelial culture were used as immunogens, and cross-reactivity could be expected since many of these antigens contain epitopes and carbohydrates residues shared with other fungal pathogens, implying that many MAbs must be screened to identify specific individual epitopes. In our case, specific purified proteins were administered with an adjuvant to boost the induction of effective antibody response, which minimizes the risk of cross-reactivity with another fungal pathogen. Likewise, the production of PAbs was chosen, considering our primary purpose, and they are cheaper, easier, and quicker to generate than MAbs, which is very expensive and requires considerable time to produce (6 and 9 months) (79, 80). Furthermore, it has been demonstrated that PAb has greater sensitivity and performance if cross-reactivity can be avoided (43). In our case, there was no reactivity with culture extracts of *C. albicans*, *A. fumigatus*, *C. neoformans*, and *Fusarium* spp., except for *P. brasiliensis* extracts, which had multiple bands. This could be due to the close relationship and genetic proximity between *H. capsulatum* and *P. brasiliensis* (81 – 83), and the potential presence of proteins with some small shared regions that polyclonal antibodies can recognize. However, there are significant differences regarding their geographic distribution, clinical characteristics, patients, and risk factors that determine the correct diagnosis procedures and therapeutic management (84 – 86).

Finally, both PAbs (*anti-Hc212_Ag* and *anti-Hc670_Ag*) were shown to be reactive against purified antigens, *H. capsulatum* yeast culture extracts, and samples from patients with a confirmed diagnosis of histoplasmosis. Previously, it has been suggested that most yeast-phase culture filtrates are characterized by a prominent slower mobility smear consistent with the presence of highly glycosylated proteins, including some with low molecular weight, like those observed at the top of the membrane in the western immunoblot (33, 70, 87, 88). This result could be attributed to the polysaccharide nature of *Hc212_Ag* and other unknown proteins homologs to *Hc670_Ag* present in *H. capsulatum*.

Considering that the major impediment to the development of a new specific immunoassay for the diagnosis of infectious disease is the identification of appropriate antigen targets, this strategy has the potential to be used as a platform to identify new diagnostic biomarkers from a broad spectrum of microbial pathogens. The antigens *Hc670_Ag* and *Hc212_Ag* specific for *H. capsulatum* are two potential antigens identified by this strategy and serve as proof of concept. However, future studies are necessary to evaluate the performance of new immunological diagnostic platforms with our anti-*Histoplasma* PAbs in terms of sensitivity and specificity with samples from patients with histoplasmosis and other fungal infections.

## MATERIALS AND METHODS

### Comparative analysis and ortholog prediction

To identify those proteins specific to *H. capsulatum*, a simple analysis of orthologs species was determined using the OrthoMCL algorithm (27) with all evaluation parameters established by default, including the following: *P* value cutoff: 1e^−5^, percent identity and percent match cutoffs: 30%, maximum weight: 100, and Markov inflation index: 1.5.

All evaluation parameters of the software were set by default. A total of 343,723 protein sequences were included in a local proteome database obtained from references strains collection of GenBank database, available at the NCBI ftp site (ftp://ftp.ncbi.nih.gov/). The collection was generated with 35 proteomes from 12 genera of fungi as follows: *Histoplasma* sp., *Emmonsia* spp., *Blastomyces* sp., *Paracoccidioides* sp., *Coccidioides* sp., *Candida* sp., *Cryptococcus* spp., *Pneumocystis* sp., *Aspergillus* sp., *Talaromyces* sp., *Fusarium* sp., *Schizophyllum* sp., and two proteomes of bacterium *Mycobacterium tuberculosis* (see Table S1).

### Prediction of secreted antigenic proteins

Complementary, secretory domain prediction was performed using SignalP 4.0 server (http://www.cbs.dtu.dk/services/SignalP) (31) to determine the presence and location of signal peptide cleavage sites and the TargetP 2.0 server (https://services.healthtech.dtu.dk/service.php?TargetP-2.0) (30) to predict other subcellular locations by N-terminal pre-sequences (mitochondrial transit peptide, chloroplast transit peptide, or thylakoid luminal transit peptide) and not consider them as secreted proteins.

All proteins identified as *Hc-specific* by OrthoMCL were processed with both amino-acid sequence-based predictors to establish the presence of signal peptides and their respective subcellular locations (mitochondrial or secretory pathway). Only those proteins with a score value above the threshold in both programs were considered as potentially secreted antigens.

### Comparative analysis with experimental data set

Databases from the previously published experimental data set were integrated into the study. The *Hc-proteins* obtained by orthological analysis were compared with a secreted proteome database obtained from pathogenic yeast-phase *H. capsulatum* culture filtrates (33), a *Histoplasma* yeast and mycelial transcriptomes database (34), and a urine-peptides database from *Histoplasma*-immunoassay-positive patients (35). Only the proteins that matched with any homologs of these experimental databases were considered for further experiments as a candidate for diagnostic biomarker.

### Expression and purification of recombinants proteins

For each *Hc_Ag* gene (*Hc110_Ag*, *Hc115_Ag*, *Hc670_Ag*, and *Hc724_Ag*), the expression vectors were obtained by GeneArt (Gene Synthesis Services, ThermoFisher Scientific, USA). The services included the full-length gene cloning into pET-100 D/TOPO expression with a polyhistidine (6× His) tag at the N-terminal region to facilitate its purification by affinity binding to a nickel-charged agarose resin (Ni-NTA). *E. coli* BL21 (DE3) were subsequently transformed and cultured. Expression of the protein in *E. coli* was induced with 0.5 mM isopropyl-β-D-thiogalactopyranoside (IPTG) at 37°C for 6 h. Then, cells were harvested by centrifugation at 5,000 rpm for 10 min at 4°C and resuspended with lysis buffer [Phosphate-buffered saline (PBS); 500 mM NaCl; 4% L-sarcosyl], followed by a freeze-thaw cycle with liquid nitrogen and incubation at 37°C for 30 min to solubilize the inclusion bodies that contain the recombinant protein. The cell extracts were centrifuged at 5,000 rpm × 10 min at 4°C to perform the recombinant His-tagged protein purification. The supernatant was passed through a HisPur Ni-NTA Spin column (ThermoFisher Scientific, USA) to bind the His-tagged *Hc_Ag* protein. Afterward, the column was washed two times with washing buffer (PBS; 500 mM NaCl; 10 mM imidazole), and the bound protein was eluted with elution buffer (PBS; 500 mM NaCl; 100 mM imidazole, pH 6.0).

Purified *Hc212_Ag* was obtained by GenScript (Protein Expression Services, GenScript Biotech Corporation, New Jersey, USA) through the mammalian Chinese Hamster Ovary expression system.

Expression of both *Hc_Ags* was confirmed by SDS-PAGE. The concentration was determined for the Bradford protein Assay. The purified recombinant proteins were stored at −80°C.

### *In vivo* immunization model

BALB/c mice were obtained from the breeding colony maintained at Corporación para Investigaciones Biológicas, CIB (Medellín, Colombia). A rapid immunization schedule previously described was adapted to recover polyclonal antisera (89). Briefly, 8–10-week-old female BALB/c mice were immunized with purified *Hc-proteins* following one intraperitoneal (i.p.) injection at day 0 (25 µg of *Hc_Ag* per injection, mixed in solution with an equal volume of Freund’s complete adjuvant) and four i.p. injections at days 7, 14, 21, and 28 (25 µg per injection) with Freund’s incomplete adjuvant. Three days after the last injection, the total blood volume of immunized mice was collected (polyclonal antisera). Additionally, spleen cells from immunized mice were stored at −80°C in Dulbecco's Modified Eagle's Medium (DMEM) supplemented with 10% Dimethyl sulfoxide (DMSO) and 20% fetal bovine serum (Gibco, Invitrogen Corporation, California, USA) for further studies.

This study followed the recommendations in the Guide for the Care and Use of Laboratory Animals of the National Institutes of Health and followed the Colombian (Law 84/1989, Resolution no. 8430/1993), European Union, and Canadian Council on Animal Care regulations. The protocol was approved by the Institutional Ethics Committee of the CIB.

### Fungal culture

Yeast culture of *Histoplasma capsulatum* (strain G186A), *Candida albicans*, *Cryptococcus neoformans*, and *Paracoccidioides brasiliensis*, and mycelium culture of *Aspergillus fumigatus* and *Fusarium* spp. were used for all studies. In addition, to the maintenance of the yeast phase of each fungus, cultures were grown at 37°C in brain heart infusion (Gibco Invitrogen Corporation, California, USA) supplemented with 100 U/mL penicillin and 100 µg/mL streptomycin (Gibco Invitrogen Corporation, California, USA), for *H. capsulatum*, Saboraud dextrose broth (SDB, BD DIFCO, Becton, Dickinson and Company, USA), for *C. albicans* and *C. neoformans*, and SDB plus 0.14% L-asparagine and 0.01% thiamine hydrochloride. Mycelium culture of *A. fumigatus* and *Fusarium* spp. was maintained at 25°C in SDB medium.

The fungal suspension was pelleted by centrifugation (1,500 rpm, 10°C for 10 min), washed two times with PBS (Gibco, Invitrogen Corporation, California, USA), and lysed using liquid nitrogen. Then, the frozen samples were resuspended in lysis buffer [50 mM Tris-HCl pH 7.5, 300 mM NaCl, 10% NP-40, and 1× protease inhibitor cocktail (Roche Holding AG, Basel, Switzerland)] and sonicated for 10 min. Finally, cell debris was pelleted by centrifugation at 5,000 rpm for 5 min, resuspended in minimum volume with lysis buffer, and stored at −20°C for subsequent analysis.

### SDS-polyacrylamide gel electrophoresis

Purified protein (*Hc_Ags*), culture extracts of fungi, and urine samples from patients with confirmed histoplasmosis were separated by SDS-polyacrylamide gel electrophoresis (SDS-PAGE). Briefly, each sample was mixed with 2× loading buffer [100 mM Tris-HCL (pH 6.8), 4% (wt/vol) SDS, 20% glycerol, 200 mM β-mercaptoethanol] and boiled for 5 min. The samples were electrophoresed on a 12% SDS-polyacrylamide gel for 90 min at 100 V at room temperature on a Mini-PROTEAN Electrophoresis system (Bio-Rad, San Diego, USA). For visualization of separated proteins, gels were stained with Coomassie blue staining buffer for 1 h, followed by washing with decoloration buffer (10% acetic acid and 5% methanol). Coomassie-stained gels were analyzed and visualized using a Trans illuminator (Molecular Imager Gel Doc XR, Bio-Rad). mPAGE Color Protein Standard (Millipore, Sigma-Aldrich, St. Louis, MO, USA) with a mixture of 10 pre-stained proteins (10–203 kDa) was used as standard size proteins in SDS-PAGE.

### Immunoblotting analysis

Immunoblot analysis was performed to evaluate the seroreactivity of serum from mice previously immunized. Briefly, the proteins resolved in SDS-PAGE were transferred onto a polyvinylidene difluoride membrane at 80 V for 90 min using the Trans-Blot Blotting System (Bio-Rad, Hercules, CA, USA). The membrane was washed twice with Tris-buffered saline (TBS) supplemented with 0.1% Tween-20, 1% methanol, blocked for 1 h with 1% skim milk in TBS, and followed by washing three times for 5 min. The membrane was probed with polyclonal serum diluted to 1:1,000 (pooled serum from immunized animals) for 2 h at room temperature by gentle shaking to perform immunoblotting. As a control, commercial monoclonal antibody anti-*C. albicans* or serum anti-Aspergillus from anonymous patients with confirmed invasive aspergillosis were used against fungal extracts (Santa Cruz Biotechnology, Texas, USA). Then, the membrane was washed three times and incubated with secondary antibody horseradish-peroxidase-labeled goat anti-IgG mouse antibody (Abcam, Cambridge, UK), dilution 1:10,000) for 1 h at room temperature. Next, the membrane was washed and subjected to chemiluminescence using 3,3′-diaminobenzidine according to the manufacturer’s specifications. Band intensity was analyzed by ChemiDoc MP Imaging System (Bio-Rad, Hercules, CA, USA). mPAGE Color Protein Standard (Millipore, Sigma-Aldrich, St. Louis, MO, USA) with a mixture of 10 pre-stained proteins (10–203 kDa) was used as standard size proteins in western blotting.

## SUPPLEMENTAL MATERIAL

Data Set S1 spectrum.00939-23-s0001.xlsx List of cluster groups with orthologous and paralogues genes obtained by OrthoMCL analysis.

Table S1 spectrum.00939-23-s0002.pdf List of proteomes by microorganism added to the local database used for the OrthoMCL analysis.

Table S2 spectrum.00939-23-s0003.pdf Comparative analysis between urine-peptides database from Histoplasma-immunoassay-positive patients and OrthoMCL analysis.

ASM does not own the copyrights to Supplemental Material that may be linked to, or accessed through, an article. The authors have granted ASM a non-exclusive, world-wide license to publish the Supplemental Material files. Please contact the corresponding author directly for reuse.
