# Supplementary material for: Identification and production of novel potential pathogen-specific biomarkers for diagnosis of histoplasmosis
Source: Microbiol Spectr. 2023 Oct 26;11(6):e00939-23. doi: 10.1128/spectrum.00939-23 (PMC10714873; doi:10.1128/spectrum.00939-23)
Supplement: Table S1 — List of proteomes by microorganism added to the local database used for the OrthoMCL analysis. [file spectrum.00939-23-s0002.pdf]

Table S1. Fungal and bacteria strains proteomes

Taxonomy name (code)*	***strain***	***Total length (Mbp)***	***Number proteins***
Histoplasma capsulatum (Hc)	*G186A*	*30*	*9254*
	H88	*37*	*9445*
	H143	*38*	*9547*
	Nam1	*33*	*9313*
Emmonsia parva (Ep)	*UAMH139*	*30*	*9562*
Emmonsia crescens (Ec)	*UAMH3008*	*30*	*9444*
Blastomyces dermatitidis (Bd)	*ATCC18188*	*73*	*11443*
Blastomyces percursus (Bp)	*EI222*	*32*	*10285*
Paracoccidioides brasiliensis (Pb)	*03*	*29*	*8427*
	18	*29*	*8390*
Paracoccidioides lutzii (Pl)	*01*	*32*	*8826*
Coccidioides immitis (Ci)	*RMSCC2394*	*28*	*10593*
Coccidioides posadasii (Cp)	*RMSCC3488*	*28*	*9964*
Candida albicans (Ca)	*SC5314*	*14*	*6030*
	WO-1	*14*	*5752*
Candida tropicalis (Ct)	*MYA3404*	*14*	*6254*
Candida parapsilosis (Cpa)	*CDC317*	*13*	*5810*
Cryptococcus neoformans var. neoformans (Cnn)	*JEC21*	*19*	*6475*
	B3501A	*19*	*13156*
Cryptococcus neoformans var. grubii (Cng)	*H99*	*18*	*7826*
Cryptococcus gatti (Cg)	*WM276*	*18*	*6561*
	2001/935-1	*17*	*6495*
	CA1873	*17*	*6634*
Pneumocystis jirovecii (Pj)	*RU7*	*8*	*3761*
Aspergillus fumigatus (Afu)	*Af293*	*29*	*19260*
Aspergillus niger (An)	*FDAARGOS311*	*35*	*11190*
Aspergillus flavus (Afl)	*NRRL3357*	*36*	*13485*
Aspergillus terreus (At)	*NIH2624*	*29*	*10401*
Talaromyces marneffei (Tm)	*ATCC18224*	*28*	*10638*
Fusarium oxysporum (Fo)	*4287*	*61*	*27347*
	Forc016	*52*	*17168*
Fusarium proliferatum (Fp)	*ET1*	*44*	*14819*
Schizophyllum commune (Sc)	*H48*	*38*	*13194*
Mycobacterium tuberculosis (Mt)	*H37Rv*	*4.4*	*3906*
	CDC1551	*4.4*	*4068*
TOTAL	***35 proteomes***		***343.723***

* Code defined by author

Supplemental Table S1. List of fungal and bacteria strains proteomes. List of proteomes by microorganism added to the local database used for the OrthoMCL analysis. For each microorganism, the total genome size (Mbp) and the total number of annotated proteins per strain are described.
