# Supplementary material for: Identification and production of novel potential pathogen-specific biomarkers for diagnosis of histoplasmosis
Source: Microbiol Spectr. 2023 Oct 26;11(6):e00939-23. doi: 10.1128/spectrum.00939-23 (PMC10714873; doi:10.1128/spectrum.00939-23)
Supplement: Table S2 — Comparative analysis between urine-peptides database from Histoplasma-immunoassay-positive patients and OrthoMCL analysis. [file spectrum.00939-23-s0003.pdf]

petide sequence	Protein Description	NCBI accesion	ID_proteom	cluster_groups	Hc_proteins cluster	Proteins_cluster	# Proteins_cluster (Hc/total)	%_Hc-proteins
LDVESADTIDAVK	polyubiquitin	154275270	EDN10288	1988	Hc03|EER45372.1<br>Hc01|EEH07231.1<br>Hc02|EGC41315.1<br>Hc04|EDN10288.1	Sc01|EFI95127.1<br>Sc01|EFJ02634.1<br>Afl1|EED55366.1<br>Tm01|XP_002151173.1<br>At01|EAU39340.1<br>Fo02|PCD32834.1<br>Fp01|CZR42472.1<br>Cg03|KIR63773.1<br>Cg02|KIR98208.1<br>An01|OWW30780.1<br>Bp01|OJD20136.1<br>Cg01|ADV24874.1<br>Cng1|AFR98116.1<br>Ec01|KKZ65431.1<br>Pl01|XP_002791169.1<br>Bd01|EGE85556.2<br>Ep01|KLJ12263.1<br>Afu1|EAL89753.1<br>Cnn1|AAW46345.1<br>Cnn2|EAL18071.1<br>Pb02|EEH45455.2<br>Pb01|EEH20847.2<br>Ct01|EER30626.1<br>Ci01|KMP00447.1<br>Ca01|AOW28729.1<br>Ca02|EEQ44747.1<br>Cpa1|CCE42351.1<br>Fo01|KNA98766.1<br>Cp01|KMM65975.1<br>Pj01|KTW32165.1	4/34	11,76%
TLSDYNIQKESTLHLVLR								
MQIFVK								
IQDKEGIPPDQQRLIFAGK	leucyl-tRNA synthetase	225554709	EEH03004	03659	Hc01|EEH03004.1<br>Hc02|EGC48352.1<br>Hc04|EDN09674.1<br>Hc03|EER40609.1	fl1|EED51244.1<br>At01|EAU33447.1<br>Afu1|EAL85549.2<br>An01|OWW36813.1<br>Tm01|XP_002153283.1<br>Bd01|EGE83589.1<br>Ec01|KKZ68928.1<br>Cp01|KMM63853.1<br>Ep01|KLJ06661.1<br>Ci01|KMP02177.1<br>Bp01|OJD22054.1<br>Pb01|EEH16845.2<br>Pb02|EEH50147.1<br>Pl01|XP_002795050.1<br>Fp01|CZR39576.1<br>Fo02|PCD37628.1<br>Fo01|KNA96512.1<br>Ct01|EER35254.1<br>Cpa1|CCE39649.1<br>Pj01|KTW31895.1<br>Ca01|AOW30275.1<br>Mt02|AAK44269.1<br>Mt01|CCP42763.1	4/27	14,81%
IDLLAVDK								
TVPSIFSSPWPDPLLSSR								
NSSTISSPPR								
NPKSRPQHLQTATTNGR	conserved hypothetical protein	240281880	EER45383	04721	Hc01|EEH07242.1<br>Hc03|EER45383.1<br>Hc02|EGC41326.1<br>Hc04|EDN10281.1	Afl1|EED55371.1<br>Afu1|EAL89748.1<br>An01|OWW30775.1<br>At01|EAU39540.1<br>Pb01|EEH20836.1<br>Pl01|XP_002791154.1<br>Pb02|EEH45443.2<br>Ec01|KKZ63456.1<br>Bp01|OJD25564.1<br>Bd01|EGE85546.1<br>Ci01|KMP00460.1<br>Cp01|KMM65963.1<br>Tm01|XP_002151166.1<br>Fo02|PCD45972.1<br>Fp01|CZR34104.1<br>Fo01|KNA95162.1	4/20	20,00%
SLEILRQR								
NAEAAGAASTKGVAGVFSYSK	DUF500 and SH3 domain-containing protein	240277865	EER41372	01167	Hc01|EEH09562.1<br>Hc04|EDN05393.1<br>Hc02|EGC49231.1<br>Hc03|EER41372.1	Afl1|EED56388.1<br>At01|EAU33632.1<br>An01|OWW37627.1<br>Afu1|EAL88641.1<br>Ep01|KLJ08046.1<br>Bd01|EGE85616.2<br>Bp01|OJD23925.1<br>Ec01|KKZ60222.1<br>Pl01|XP_015701596.1<br>Tm01|XP_002144761.1<br>Pb02|EEH45030.2<br>Pb01|EEH19574.2<br>Ci01|KMP01186.1<br>Cp01|KMM65256.1<br>Fp01|CZR36585.1<br>Fo02|PCD43606.1<br>Fo01|KNB03852.1<br>Ct01|EER34769.1<br>Ca02|EEQ45902.1<br>Ca01|AOW27508.1<br>Sc01|EFJ04083.1<br>Cpa1|CCE43313.1<br>Cnn1|AAW43941.1<br>Cnn2|EAL20065.1<br>Cng1|AFR96209.2<br>Cg01|ADV23105.1<br>Cg03|KIR63956.1<br>Cg02|KIR97013.1<br>Cg02|KIR99840.1<br>Cg01|ADV25802.1<br>Cg03|KIR58482.1<br>Cnn1|AAW47052.2<br>Cnn2|EAL17297.1<br>[illegible]	4/39	10,26%
IGDRTGIFPSNYVDASNL								

petide sequence	Protein Description	NCBI accesion	ID_proteom	cluster_groups	Hc_proteins cluster	Proteins_cluster	# Proteins_cluster (Hc/total)	%_Hc-proteins
GITGEYIHILVKPDMEK	5-oxoprolinase	240280678	EER44182	00343	Hc03|EER44182.1<br>Hc01|EEH09061.1<br>Hc02|EGC42379.1<br>Hc04|EDN08864.1<br>Hc01|EEH08667.1<br>Hc02|EGC41968.1<br>Hc01|EEH08668.1<br>Hc03|EER43446.1	Afl1|EED44907.1<br>Afl1|EED44932.1<br>Afl1|EED56562.1<br>Afl1|EED57513.1<br>An01|OWW28142.1<br>An01|OWW28966.1<br>An01|OWW31707.1<br>An01|OWW34653.1<br>An01|OWW35378.1<br>An01|OWW35998.1<br>At01|EAU32239.1<br>At01|EAU39548.1<br>At01|EAU39587.1<br>Bd01|EGE84974.1<br>Bd01|EGE85158.1<br>Bp01|OJD22429.1<br>Bp01|OJD24359.1<br>Cng1|AFR94606.1<br>Cng1|AFR97967.1<br>Cng1|AFR94607.2<br>Cng1|AFR95371.2<br>Cng1|AFR97968.2<br>Cnn1|AAW42251.1<br>Cnn1|AAW43748.1<br>Cnn1|AAW42254.1<br>Cnn1|AAW43528.2<br>Cnn1|AAW43746.1<br>Cnn2|EAL20409.1<br>Cnn2|EAL20727.1<br>Cnn2|EAL21755.1<br>Cnn2|EAL20410.1<br>Cnn2|EAL21756.1<br>Ec01|KKZ60387.1<br>Ec01|KKZ61732.1	8/77	10,39%
NHTSISVQDRDPPIR	mannosyl-oligosaccharide 1,2-alpha- mannosidase	150412285	EDN07672	00938	Hc02|EGC42817.1<br>Hc01|EEH10603.1<br>Hc04|EDN07672.1<br>Hc03|EER42723.1<br>Hc03|EER36421.1<br>Hc01|EEH06244.1<br>Hc04|EDN04787.1<br>Hc02|EGC48997.1	At01|EAU36178.1<br>At01|EAU39466.1<br>Bd01|EGE77192.1<br>Bd01|EGE79766.1<br>Bp01|OJD27023.1<br>Bp01|OJD27304.1<br>Ec01|KKZ58585.1<br>Ec01|KKZ66787.1<br>Ep01|KLJ10937.1<br>Ep01|KLJ12326.1<br>Fo01|KNA94342.1<br>Fo01|KNA94343.1<br>Afl1|EED56472.1<br>Afu1|EAL88534.1<br>An01|OWW27990.1<br>Tm01|XP_002148939.1<br>Ci01|KMP02442.1<br>Cp01|KMM68355.1<br>Pb01|KGY15697.1<br>Pb02|EEH47977.1<br>Pl01|XP_002796910.1<br>Fp01|CZR34876.1<br>Fo02|PCD45551.1<br>Ca02|EEQ42796.1<br>Ca01|AOW26050.1<br>Cpa1|CCE40999.1<br>Ct01|EER31569.1<br>Pj01|KTW28218.1<br>Cg01|ADV22676.1<br>Cg03|KIR58826.1<br>Cng1|AFR95738.1<br>Cg02|KIR99692.1<br>Cnn1|AAW43838.1<br>Cnn2|EAL20535.1	8/44	18,18%
ITHSWVLQSLTRRAW	ADP-ribosylation factor	150408441	EDN03982	00785	Hc03|EER42346.1<br>Hc02|EGC43406.1<br>Hc04|EDN03984.1<br>Hc03|EER42345.1<br>Hc02|EGC43405.1<br>Hc01|EEH08370.1<br>Hc04|EDN03982.1	Fo01|KNA94253.1<br>Fo01|KNA94254.1<br>Tm01|XP_002151474.1<br>Tm01|XP_002151475.1<br>Afl1|EED49980.1<br>At01|EAU39012.1<br>An01|OWW28322.1<br>Afu1|EBA27409.1<br>Tm01|XP_002151476.1<br>Pl01|XP_015700697.1<br>Pb02|EEH46734.2<br>Pb01|EEH17859.2<br>Bp01|OJD26316.1<br>Bd01|KMW66692.1<br>Ep01|KLJ06327.1<br>Ec01|KKZ64459.1<br>Ci01|KMP04159.1<br>Cp01|KMM68037.1<br>Afl1|EED49982.1<br>At01|EAU39010.1<br>Pb01|EEH17860.2<br>Pb02|EEH46735.1<br>Pl01|XP_002790403.1<br>Afu1|EAL90501.1<br>Bp01|OJD26315.1<br>Bd01|EGE78278.1<br>Ec01|KKZ64458.1<br>Ep01|KLJ06328.1<br>An01|OWW28323.1<br>Cp01|KMM68036.1<br>Fo02|PCD45494.1<br>Fp01|CZR34932.1<br>Cpa1|CCE44752.1<br>Ct01|EER33522.1	7/52	13,46%
KQTNYVSGLFRNSRT	predicted protein	239613150				Blastomyces dermatitidis		
AISDLSWSPDGK	histone transcription regulator slm9	240276397	EER39909	1829	Hc03|EER39909.1<br>Hc04|EDN03592.1<br>Hc01|EEH08017.1<br>Hc02|EGC43052.1	Cnn1|AAW45342.1<br>Cnn1|AAW45343.1<br>Afl1|EED54859.1<br>An01|OWW28165.1<br>Afu1|EAL90308.1<br>At01|EAU35982.1<br>Tm01|XP_002151632.1<br>Pl01|XP_015703208.1<br>Pb02|EEH45698.2<br>Pb01|EEH21052.2<br>Bp01|OJD23578.1<br>Ec01|KKZ57979.1<br>Bd01|EGE80790.1<br>Ep01|KLJ10650.1<br>Ci01|KMP09638.1<br>Cp01|KMM71504.1<br>Fo02|PCD43826.1<br>Fp01|CZR36814.1<br>Fo01|KNB04224.1<br>Pj01|KTW31170.1<br>Ca01|AOW27087.1<br>Cpa1|CCE43703.1<br>Ct01|EER34220.1<br>Ca02|EEQ45485.1<br>Cng1|AFR96888.1<br>Cg03|KIR62380.1<br>Cg02|KIR98356.1<br>Cg01|ADV24167.1<br>Cnn2|EAL19183.1<br>Sc01|EFI94039.1	4/34	11,76%
SWSPDG								

petide sequence	Protein Description	NCBI accesion	ID_proteom	cluster_groups	Hc_proteins cluster	Proteins_cluster	# Proteins_cluster (Hc/total)	%_Hc-proteins
GWDQTGSNGANL	hypothetical protein HCAG 00002	150406597	EDN02138	00005	Hc01|EEH04492.1<br>Hc01|EEH09904.1<br>Hc01|EEH04783.1<br>Hc01|EEH06216.1<br>Hc01|EEH05554.1<br>Hc01|EEH09472.1<br>Hc02|EGC40813.1<br>Hc02|EGC48971.1<br>Hc02|EGC44370.1<br>Hc02|EGC48561.1<br>Hc02|EGC49990.1<br>Hc02|EGC50037.1<br>Hc03|EER36447.1<br>Hc03|EER37123.1<br>Hc03|EER43728.1<br>Hc03|EER44451.1<br>Hc04|EDN02138.1<br>Hc04|EDN04810.1<br>Hc04|EDN04400.1<br>Hc04|EDN05861.1<br>Hc04|EDN08417.1<br>Hc04|EDN08473.1<br>Hc03|EER39527.1<br>Hc02|EGC46681.1<br>Hc04|EDN07414.1<br>Hc01|EEH11692.1<br>Hc01|EEH07352.1<br>Hc02|EGC41439.1<br>Hc04|EDN10605.1<br>Hc02|EGC42705.1<br>Hc03|EER42607.1<br>Hc01|EEH04071.1<br>Hc04|EDN11365.1<br>[illegible]	Afl1|EED44759.1<br>Afl1|EED52734.1<br>Afl1|EED44875.1<br>Afl1|EED56984.1<br>Afl1|EED45470.1<br>Afl1|EED46489.1<br>Afl1|EED50679.1<br>Afl1|EED45540.1<br>Afl1|EED49992.1<br>Afl1|EED49844.1<br>Afl1|EED50421.1<br>Afl1|EED52999.1<br>Afl1|EED54099.1<br>Afl1|EED53116.1<br>Afl1|EED53300.1<br>Afu1|EAL84263.1<br>Afu1|EAL85448.1<br>Afu1|EAL86889.1<br>Afu1|EAL84338.1<br>Afu1|EAL93753.1<br>Afu1|EAL84797.1<br>Afu1|EAL86188.1<br>Afu1|EAL93446.1<br>Afu1|EAL86652.1<br>Afu1|EAL92404.2<br>Afu1|EAL88065.2<br>Afu1|EBA27373.1<br>An01|OWW28338.1<br>An01|OWW29638.1<br>An01|OWW28995.1<br>An01|OWW33348.1<br>An01|OWW36174.1<br>An01|OWW29964.1<br>[illegible]	72/1054	6,83%
RLGLSALIRE	glycogen debranching enzyme	240273359	EER36880	00461	Hc04|EDN06738.1<br>Hc01|EEH06405.1<br>Hc02|EGC49156.1<br>Hc03|EER36880.1<br>Hc02|EGC49157.1<br>Hc01|EEH06407.1<br>Hc03|EER36882.1<br>Hc04|EDN06739.1	Fo01|KNB07054.1<br>Fo01|KNB07055.1<br>Afl1|EED57438.1<br>At01|EAU35326.1<br>An01|OWW31787.1<br>Afu1|EAL87926.1<br>Tm01|XP_002148450.1<br>Ec01|KKZ65058.1<br>Ep01|KLJ07169.1<br>Fp01|CZR37037.1<br>Fo02|PCD44046.1<br>Pb01|EEH16472.1<br>Pb02|EEH43199.2<br>Ci01|KMP09327.1<br>Cp01|KMM71193.1<br>Bp01|OJD20475.1<br>Bd01|KMW67998.1<br>Pj01|KTW32045.1<br>Cpa1|CCE43776.1<br>Ca01|AOW29254.1<br>Ca02|EEQ44997.1<br>Ct01|EER32280.1<br>Sc01|EFI98261.1<br>Cg02|KIR96655.1<br>Cg01|ADV24562.1<br>Cg03|KIR57828.1<br>Cnn1|AAW45872.1<br>Cnn2|EAL18619.1<br>Cng1|AFR97337.2<br>Pl01|XP_015702671.1<br>Afl1|EED57439.1<br>Afu1|EAL87925.1<br>At01|EAU35327.1<br>[illegible]	8/68	11,76%
NLLTCMSR	predicted protein	150408639	EDN04180	00002	Hc01|EEH08174.1<br>Hc01|EEH08224.1<br>Hc04|EDN02137.1<br>Hc04|EDN04710.1<br>Hc04|EDN07506.1<br>Hc04|EDN09865.1<br>Hc04|EDN10980.1<br>Hc04|EDN03895.1<br>Hc04|EDN04666.1<br>Hc04|EDN02414.1<br>Hc04|EDN08380.1<br>Hc04|EDN11161.1<br>Hc04|EDN06998.1<br>Hc04|EDN11419.1<br>Hc04|EDN09821.1<br>Hc04|EDN10535.1<br>Hc04|EDN05359.1<br>Hc04|EDN08398.1<br>Hc04|EDN04763.1<br>Hc04|EDN05161.1<br>Hc04|EDN06633.1<br>Hc04|EDN11267.1<br>Hc04|EDN10552.1<br>Hc04|EDN05478.1<br>Hc04|EDN02167.1<br>Hc04|EDN11415.1<br>Hc04|EDN02183.1<br>Hc04|EDN02638.1<br>Hc04|EDN05001.1<br>Hc04|EDN05516.1<br>Hc04|EDN06571.1<br>Hc04|EDN06868.1<br>Hc04|EDN06935.1<br>[illegible]	At01|EAU29721.1<br>At01|EAU30981.1<br>At01|EAU32284.1<br>At01|EAU32866.1<br>At01|EAU33186.1<br>At01|EAU30143.1<br>At01|EAU35995.1<br>Bd01|EGE78131.2<br>Bd01|KMW68256.1<br>Bd01|EGE86177.2<br>Bd01|KMW68669.1<br>Bd01|KMW69004.1<br>Bd01|KMW69230.1<br>Bd01|KMW69080.1<br>Bd01|EGE79076.2<br>Bd01|EGE81043.1<br>Bd01|EGE79139.2<br>Bd01|KMW68378.1<br>Bd01|EGE82461.2<br>Bd01|EGE86174.2<br>Bd01|KMW68867.1<br>Bd01|KMW67939.1<br>Bd01|KMW69634.1<br>Bd01|KMW66926.1<br>Bd01|KMW69183.1<br>Bd01|KMW67281.1<br>Bd01|EGE86559.1<br>Bd01|KMW69162.1<br>Bd01|EGE87087.1<br>Bd01|KMW69489.1<br>Bd01|KMW69490.1<br>Bd01|EGE87131.1<br>Bd01|KMW69403.1<br>[illegible]	579/1595	**36,30%**
NSFVNLALPFFSFIDPIASPMDK	ubiquitin-activating enzyme	240274939	EER38454	0542	Hc02|EGC47601.1<br>Hc01|EEH06787.1<br>Hc03|EER38453.1<br>Hc02|EGC47602.1<br>Hc01|EEH06789.1<br>Hc03|EER38454.1<br>Hc04|EDN03141.1	Afl1|EED54252.1<br>Afu1|EAL93884.1<br>An01|OWW37245.1<br>Tm01|XP_002147937.1<br>At01|EAU37426.1<br>Bp01|OJD22322.1<br>Ci01|KMP00146.1<br>Pb02|EEH47327.1<br>Pb01|EEH18389.2<br>Cp01|KMM66261.1<br>Bd01|KMW67727.1<br>Ep01|KLJ08001.1<br>Ec01|KKZ67571.1<br>Pl01|XP_015703053.1<br>Fo02|PCD44111.1<br>Fp01|CZR37105.1<br>Fo01|KNB07163.1<br>Pj01|KTW27131.1<br>Sc01|EFJ02093.1<br>Cnn1|AAW46647.2<br>Cng1|AFR94238.2<br>Cnn2|EAL18781.1<br>Cg02|KIS02525.1<br>Ct01|EER31188.1<br>Ca02|EEQ46846.1<br>Ca01|AOW30413.1<br>Cg01|ADV21465.1<br>Cg03|KIR59482.1<br>Cpa1|CCE41149.1<br>Afl1|EED54271.1<br>At01|EAU37413.1<br>An01|OWW37234.1<br>Afu1|EAL93873.1<br>[illegible]	7/65	10,77%
LNNSEPR								

petide sequence	Protein Description	NCBI accesion	ID_proteom	cluster_groups	Hc_proteins cluster	Proteins_cluster	# Proteins_cluster (Hc/total)	%_Hc-proteins
RQFVDSLSEGLGEQGKG	conserved hypothetical protein	240281152	EER44655	05017	Hc03|EER44655.1<br>Hc02|EGC45661.1<br>Hc04|EDN07762.1<br>Hc01|EEH10724.1	Afl1|EED57997.1<br>Afu1|EAL92761.1<br>An01|OWW29351.1<br>At01|EAU38412.1<br>Tm01|XP_002153509.1<br>Bd01|EGE77276.2<br>Ec01|KKZ67046.1<br>Pl01|XP_015700892.1<br>Ep01|KLJ13286.1<br>Pb01|EEH19214.2<br>Pb02|EEH48059.1<br>Bp01|OJD09448.1<br>Ci01|KMP05964.1<br>Cp01|KMM69615.1	4/18	22,22%
RGEHKYDPVVTLQPFGNKA	TdiA	239614048				Blastomyces dermatitidis		
SKHQHFVGLSFDTISST	xaa-proaminopeptidase	240273135	EER36658	00113	Hc04|EDN10027.1<br>Hc01|EEH05721.1<br>Hc02|EGC49445.1<br>Hc03|EER41579.1<br>Hc01|EEH10445.1<br>Hc02|EGC44919.1<br>Hc04|EDN06389.1<br>Hc03|EER36658.1<br>Hc01|EEH08670.1<br>Hc03|EER43448.1<br>Hc02|EGC41970.1<br>Hc04|EDN05281.1<br>Hc01|EEH09839.1<br>Hc04|EDN05810.1<br>Hc02|EGC44313.1<br>Hc03|EER38181.1	Fo01|KNB10186.1<br>Fo01|KNB10187.1<br>Fo01|KNB10188.1<br>Fo01|KNB10189.1<br>Fo01|KNB10190.1<br>Fo01|KNB10191.1<br>Fo01|KNB10300.1<br>Fo01|KNB10301.1<br>Tm01|XP_002145079.1<br>Tm01|XP_002145080.1<br>Afl1|EED48681.1<br>At01|EAU33225.1<br>An01|OWW37949.1<br>Afu1|EAL88769.2<br>Tm01|XP_002144715.1<br>Ep01|KLJ09957.1<br>Ec01|KKZ66702.1<br>Bd01|EGE77555.2<br>Bp01|OJD28084.1<br>Pl01|XP_015701057.1<br>Pb02|EEH47511.2<br>Pb01|EEH19479.1<br>Cp01|KMM65313.1<br>Ci01|KMP01130.1<br>Fp01|CZR36317.1<br>Fo02|PCD43352.1<br>Fo01|KNB03434.1<br>Cpa1|CCE44634.1<br>Pj01|KTW30512.1<br>Ca01|AOW28384.1<br>Ca02|EEQ44373.1<br>Cg01|ADV21286.1<br>Ct01|EER33649.1<br>Cnn1|AAW42477.1	16/145	11,03%
DIEFLIPIQCSILSERR								
LDALRVPLTHLLAVR								
RSKERSWLMLFVYDRS	priB	240276009	EER39522	00061	Hc01|EEH10922.1<br>Hc02|EGC45890.1<br>Hc03|EER42955.1<br>Hc02|EGC45891.1<br>Hc01|EEH10923.1<br>Hc03|EER42956.1<br>Hc04|EDN07948.1<br>Hc03|EER38722.1<br>Hc01|EEH03554.1<br>Hc02|EGC47877.1<br>Hc04|EDN03345.1<br>Hc03|EER38721.1<br>Hc03|EER39522.1<br>Hc02|EGC46676.1<br>Hc01|EEH11687.1<br>Hc04|EDN07411.1<br>Hc03|EER38313.1<br>Hc01|EEH06655.1<br>Hc04|EDN03034.1<br>Hc02|EGC47460.1	Cg01|ADV19990.1<br>Cg01|ADV25736.1<br>Cg02|KIR96953.1<br>Cg02|KIS00240.1<br>Cg03|KIR58421.1<br>Cg03|KIR68920.1<br>Cng1|AFR92972.2<br>Cng1|AFR98577.2<br>Cng1|AFR93274.2<br>Cng1|AGV15392.1<br>Cnn1|AAW41378.2<br>Cnn1|AAW47089.2<br>Cnn1|ALO60369.1<br>Cnn2|EAL17240.1<br>Cnn2|EAL23034.1<br>Fo01|KNA97029.1<br>Fo01|KNA97030.1<br>Fo01|KNA97031.1<br>Fo01|KNA97032.1<br>Fo01|KNB01187.1<br>Fo01|KNB01188.1<br>Fo01|KNB01324.1<br>Fo01|KNB03221.1<br>Fo01|KNB08176.1<br>Fo01|KNB03222.1<br>Fo01|KNB18205.1<br>Fo01|KNB18206.1<br>Fo01|KNB18207.1<br>Fo02|PCD30639.1<br>Fo02|PCD42879.1<br>Fp01|CZR36149.1<br>Fp01|CZR40927.1<br>Fp01|CZR46005.1<br>Afl1|EED45976.1	20/239	8,37%
YFDSIPGAVTRKEDR	methionine sulfoxide reductase msrB	150411212	EDN06600	02112	Hc04|EDN06600.1<br>Hc01|EEH09566.1<br>Hc03|EER41368.1<br>Hc02|EGC49227.1	Afl1|EED56379.1<br>Afu1|EAL88637.2<br>An01|OWW37619.1<br>At01|EAU33638.1<br>Tm01|XP_002144765.1<br>Fo01|KNB03855.1<br>Fp01|CZR36588.1<br>Fo02|PCD43609.1<br>Ec01|KKZ60217.1<br>Ci01|KMP01194.1<br>Cp01|KMM65250.1<br>Bd01|EGE85612.2<br>Bp01|OJD10177.1<br>Ep01|KLJ08043.1<br>Pl01|XP_002797457.2<br>Pb01|KGY15782.1<br>Pb02|EEH47424.1<br>Sc01|EFI98243.1<br>Cng1|AFR93517.2<br>Cnn1|AAW41684.2<br>Cnn2|EAL22852.1<br>Cg01|ADV20022.1<br>Cg03|KIR59783.1<br>Cg02|KIS00881.1<br>Cpa1|CCE40971.1<br>Ca02|EEQ43038.1<br>Ca01|AOW25800.1<br>Ct01|EER31622.1<br>Mt02|AAK47063.1<br>Mt01|CCP45472.1	4/34	11,76%
TVIVITRRSLEPAR	predicted protein	239611175				Blastomyces dermatitidis		

petide sequence	Protein Description	NCBI accesion	ID_proteom	cluster_groups	Hc_proteins cluster	Proteins_cluster	# Proteins_cluster (Hc/total)	%_Hc-proteins
NWMRELMLARALK	SAM and PH domain-containing protein	225555719	EEH04010	04716	Hc01|EEH04010.1<br>Hc02|EGC45344.1<br>Hc04|EDN11430.1<br>Hc03|EER40053.1	Afl1|EED54940.1<br>Afu1|EAL88557.2<br>Tm01|XP_002150440.1<br>Bp01|OJD28147.1<br>Ec01|KKZ66641.1<br>Bd01|EGE82597.2<br>At01|EAU35535.1<br>Pb01|EEH20612.2<br>Pb02|EEH45199.1<br>Pl01|XP_015700119.1<br>Ci01|KMP06302.1<br>Cp01|KMM69278.1<br>An01|OWW36691.1<br>Fo02|PCD37700.1<br>Fo01|KNA96643.1<br>Fp01|CZR39647.1	4/20	20,00%
PAPFCGTCNPISGK	YPS-3 yeast phase-specific protein	348156	EEH04399	00806	Hc01|EEH04399.1<br>Hc03|EER41904.1<br>Hc02|EGC43996.1<br>Hc04|EDN09203.1<br>Hc01|EEH06922.1<br>Hc02|EGC47746.1<br>Hc03|EER38598.1<br>Hc04|EDN03248.1<br>Hc02|EGC43366.1<br>Hc01|EEH08333.1<br>Hc04|EDN05128.1<br>Hc01|EEH04749.1<br>Hc03|EER37085.1<br>Hc04|EDN03955.1<br>Hc02|EGC40778.1	Ct01|EER30652.1<br>Ct01|EER30654.1<br>Ct01|EER35213.1<br>Ct01|EER31209.1<br>Ct01|EER31218.1<br>Ct01|EER31221.1<br>Mt01|CCP44519.1<br>Mt01|CCP44685.1<br>Mt02|AAK46069.1<br>Mt02|AAK46241.1<br>Afu1|EAL93308.1<br>At01|EAU34894.1<br>An01|OWW34020.1<br>Ep01|KLJ10675.1<br>Ec01|KKZ65810.1<br>Pl01|XP_002789111.2<br>Pb01|EEH19617.1<br>Pb02|KGM91401.1<br>Ci01|KMP05916.1<br>Cp01|KMM69660.1<br>Bp01|OJD22974.1<br>Bd01|EGE78732.1<br>Ec01|KKZ61504.1<br>Bd01|EGE86383.1<br>Ep01|KLJ09265.1<br>Pl01|XP_002794451.1<br>Pb01|EEH17892.1<br>Pb02|EEH46769.1<br>Bp01|OJD20817.1<br>Ec01|KKZ68597.1<br>Ca01|AOW28806.1<br>Ca02|EEQ45444.1<br>Cpa1|CCE44489.1<br>[illegible]	15/51	29,41%
KLDGAVPFKVQTRM	predicted protein	150409767	EDN05207	00215	Hc01|EEH05486.1<br>Hc04|EDN02759.1<br>Hc01|EEH09326.1<br>Hc04|EDN08594.1<br>Hc03|EER43902.1<br>Hc02|EGC49844.1<br>Hc04|EDN05207.1<br>Hc01|EEH03224.1<br>Hc02|EGC45141.1<br>Hc03|EER41255.1<br>Hc02|EGC47139.1<br>Hc03|EER36356.1	Afl1|EED45254.1<br>Afl1|EED54104.1<br>An01|OWW27560.1<br>An01|OWW37371.1<br>Tm01|XP_002147181.1<br>Tm01|XP_002147182.1<br>Fo02|PCD26430.1<br>Fo01|KNB08826.1<br>Afl1|EED45413.1<br>An01|OWW35510.1<br>At01|EAU29573.1<br>Fo01|KNB03084.1<br>Fo02|PCD43106.1<br>Ci01|KMP05511.1<br>Cp01|KMM70806.1<br>Ec01|KKZ61233.1<br>Fp01|CZR36040.1<br>Ep01|KLJ11988.1<br>Bp01|OJD22539.1<br>Afu1|EAL89915.1<br>Tm01|XP_002144725.1<br>Afl1|EED45662.1<br>At01|EAU36032.1<br>Fp01|CZR36163.1<br>Fo01|KNB03235.1<br>An01|OWW35820.1<br>Fo02|PCD23604.1<br>Ec01|KKZ65324.1<br>Ep01|KLJ10895.1<br>Tm01|XP_002151116.1<br>Bp01|OJD22991.1<br>Afl1|EED47542.1<br>An01|OWW37955.1<br>[illegible]	12/98	12,24%
MKPFTEEETIK	phenol monooxygenase	239609148				Blastomyces dermatitidis		
KCPTLSPENKN	predicted protein	150407363	EDN02904	03549	Hc01|EEH06516.1<br>Hc03|EER40696.1<br>Hc04|EDN02904.1<br>Hc02|EGC47317.1	Afl1|EED54630.1<br>At01|EAU39114.1<br>An01|OWW32790.1<br>Afu1|EAL90614.1<br>Tm01|XP_002146263.1<br>Bd01|EGE81802.2<br>Pl01|XP_002790432.1<br>Ep01|KLJ11126.1<br>Ec01|KKZ65344.1<br>Bp01|OJD27525.1<br>Pb01|EEH17830.1<br>Pb02|EEH46705.1<br>Cp01|KMM67882.1<br>Ci01|KMP04001.1<br>Fo01|KNB06858.1<br>Fo02|PCD43953.1<br>Fp01|CZR36941.1<br>Pj01|KTW32402.1<br>Sc01|EFI95119.1<br>Cg02|KIS01205.1<br>Cng1|AFR92391.2<br>Cg01|ADV20298.1<br>Cnn2|EAL23585.1<br>Cnn1|AAW41332.2	4/28	14,29%
LLFVGSNSAPGR	predicted protein	154280483	EDN06621	Singletons - Hc04				
TLPDDHILQEAK								

petide sequence	Protein Description	NCBI accesion	ID_proteom	cluster_groups	Hc_proteins cluster	Proteins_cluster	# Proteins_cluster (Hc/total)	%_Hc-proteins
KLNMRVARWRE	conserved hypothetical protein	150411980	EDN07368	00621	Hc02|EGC47525.1<br>Hc03|EER38378.1<br>Hc01|EEH06714.1<br>Hc01|EEH11644.1<br>Hc03|EER39473.1<br>Hc04|EDN07368.1<br>Hc02|EGC46632.1<br>Hc04|EDN03081.1<br>Hc04|EDN03082.1	Bd01|EGE84844.2<br>Bd01|KMW68456.1<br>Fo01|KNB04145.1<br>Fo01|KNB04146.1<br>Tm01|XP_002146800.1<br>Tm01|XP_002148766.1<br>Tm01|XP_002148767.1<br>Afl1|EED45728.1<br>At01|EAU31879.1<br>An01|OWW33465.1<br>Afu1|EAL91543.1<br>Pl01|XP_015701644.1<br>Bp01|OJD20271.1<br>Pb02|EEH49355.2<br>Ep01|KLJ07076.1<br>Cp01|KMM65826.1<br>Ci01|KMP00598.1<br>Pb01|EEH22589.2<br>Ec01|KKZ60111.1<br>Fo02|PCD43784.1<br>Fp01|CZR36769.1<br>Pj01|KTW27160.1<br>Cg02|KIR98138.1<br>Cng1|AFR93988.2<br>Cg03|KIR63701.1<br>Cg01|ADV24888.1<br>Cnn2|EAL18143.1<br>Cnn1|AAW46152.2<br>Sc01|EFI94384.1<br>Ct01|EER33495.1<br>Ca01|AOW28454.1<br>Ca02|EEQ44479.1<br>Cpa1|CCE42063.1<br>Afl1|EED50735.1	9/60	15,00%
ISITDPANDR	conserved hypothetical protein	225561657	EEH09937	04861	Hc01|EEH09937.1<br>Hc03|EER38774.1<br>Hc02|EGC44405.1<br>Hc04|EDN05893.1	Afu1|EAL91257.1<br>At01|EAU33910.1<br>An01|OWW33537.1<br>Bd01|EGE80985.1<br>Ec01|KKZ66462.1<br>Tm01|XP_002145096.1<br>Cp01|KMM71885.1<br>Ci01|KMP08869.1<br>Ep01|KLJ06761.1<br>Bp01|OJD27774.1<br>Pb01|EEH15769.2<br>Pb02|EEH50813.2<br>Pl01|XP_002795865.2<br>Fo02|PCD41649.1<br>Fp01|CZR38733.1	4/19	21,05%
RVATSILRA	cysteine synthase B	239613737				Blastomyces dermatitidis		
LSIQATLIR	conserved hypothetical protein	240275767	EER39280	1305	Hc02|EGC46447.1<br>Hc01|EEH11467.1<br>Hc03|EER39280.1<br>Hc04|EDN07194.1<br>Hc03|EER39281.1	Fo01|KNA96609.1<br>Fo01|KNA96610.1<br>Fo01|KNA96611.1<br>Afl1|EED56241.1<br>An01|OWW37784.1<br>At01|EAU33763.1<br>Afu1|EAL87595.1<br>Tm01|XP_002144613.1<br>Ec01|KKZ64878.1<br>Pb01|EEH19348.2<br>Ep01|KLJ10160.1<br>Pb02|EEH47656.2<br>Bd01|EGE77776.1<br>Cp01|KMM65455.1<br>Pl01|XP_015701018.1<br>Bp01|OJD27400.1<br>Ci01|KMP00982.1<br>Fo02|PCD37680.1<br>Fp01|CZR39629.1<br>Ca01|AOW29634.1<br>Sc01|EFJ03495.1<br>Cg03|KIR58136.1<br>Cng1|AFR99043.2<br>Cg01|ADV25317.1<br>Cnn1|AAW45123.2<br>Cnn2|EAL17745.1<br>Pj01|KTW30413.1<br>Ct01|EER30875.1<br>Ca02|EEQ46244.1<br>Cpa1|CCE40118.1<br>Ct01|EER30873.1<br>Cg02|KIR96401.1	5/37	13,51%
LSIQA								
AENTAESPESEDVKVK	DNA binding protein URE-B1	240276203	EER39715	1122	Hc01|EEH08178.1<br>Hc01|EEH08228.1<br>Hc02|EGC43241.1<br>Hc04|EDN03803.1<br>Hc03|EER39715.1	Bd01|EGE79301.1<br>Bd01|KMW66969.1<br>Afl1|EED55409.1<br>At01|EAU35793.1<br>An01|OWW30735.1<br>Afu1|EAL89711.1<br>Tm01|XP_002151216.1<br>Bp01|OJD22722.1<br>Ec01|KKZ67992.1<br>Pb02|EEH45502.1<br>Pb01|KGY15381.1<br>Pl01|XP_002795156.1<br>Cp01|KMM65016.1<br>Ci01|KMP01441.1<br>Ep01|KLJ12683.1<br>Fo02|PCD46065.1<br>Fo01|KNA95337.1<br>Fp01|CZR33910.1<br>Pj01|KTW29969.1<br>Sc01|EFI94524.1<br>Ca02|EEQ45750.1<br>Cpa1|CCE43528.1<br>Ct01|EER34446.1<br>Ca01|AOW27355.1<br>Cng1|AFR97151.1<br>Cnn1|AAW45380.2<br>Cnn2|EAL19428.1<br>Cg02|KIR98626.1<br>Cg01|ADV24184.1<br>Cg03|KIR59285.1<br>Bp01|OJD23673.1<br>An01|OWW30736.1<br>Ca02|EEQ45752.1<br>Ca02|EEQ45751.1	5/39	12,82%

petide sequence	Protein Description	NCBI accession	ID_proteom	cluster_groups	Hc_proteins cluster	Proteins_cluster	# Proteins_cluster (Hc/total)	%_Hc-proteins
ADPFATTPSKPR0								
MFYFDSEFVGPPR	predicted protein	150414072	EDN09437	05238	Hc04|EDN09437.1<br>Hc01|EEH04145.1<br>Hc02|EGC48357.1<br>Hc01|EEH03007.1<br>Hc03|EER42161.1<br>Hc02|EGC43734.1<br>Hc03|EER40420.1	Bd01|EGE86184.2<br>Bp01|OJD22518.1<br>Pl01|XP_015700594.1<br>Pb02|EEH44665.2<br>Pb01|KGY15563.1<br>Ep01|KLJ06008.1<br>Ec01|KKZ65985.1<br>Cg01|ADV24578.1<br>Cg03|KIR57842.1	7/16	43,75%
LLWGGAQQER								
RFSIKSCLFPHAKK	conserved hypothetical protein	150412949	EDN08366	00033	Hc04|EDN03543.1<br>Hc04|EDN03549.1<br>Hc04|EDN04232.1<br>Hc04|EDN04611.1<br>Hc04|EDN04927.1<br>Hc04|EDN04930.1<br>Hc04|EDN04947.1<br>Hc04|EDN05349.1<br>Hc04|EDN07500.1<br>Hc04|EDN10537.1<br>Hc04|EDN10543.1<br>Hc04|EDN03545.1<br>Hc04|EDN03551.1<br>Hc04|EDN04227.1<br>Hc04|EDN04235.1<br>Hc04|EDN04239.1<br>Hc04|EDN04608.1<br>Hc04|EDN04610.1<br>Hc04|EDN04914.1<br>Hc04|EDN04922.1<br>Hc04|EDN04935.1<br>Hc04|EDN04936.1<br>Hc04|EDN04941.1<br>Hc04|EDN04944.1<br>Hc04|EDN04949.1<br>Hc04|EDN05266.1<br>Hc04|EDN05351.1<br>Hc04|EDN05464.1<br>Hc04|EDN05497.1<br>Hc04|EDN05500.1<br>Hc04|EDN05506.1<br>Hc04|EDN06546.1<br>Hc04|EDN06557.1<br>Hc04|EDN06580.1	Afu1|EAL84881.1<br>Afu1|EAL87107.1<br>Afu1|EAL92048.1<br>Afu1|EAL94109.1<br>Afu1|EAL85503.1<br>Afu1|EAL91105.1<br>At01|EAU29224.1<br>At01|EAU29404.1<br>At01|EAU31662.1<br>At01|EAU32783.1<br>At01|EAU32784.1<br>At01|EAU34001.1<br>At01|EAU34003.1<br>At01|EAU34004.1<br>At01|EAU36851.1<br>At01|EAU36852.1<br>At01|EAU37739.1<br>At01|EAU39627.1<br>At01|EAU29403.1<br>At01|EAU37740.1<br>Bp01|OJD19313.1<br>Bp01|OJD22346.1<br>Cng1|AFR92135.2<br>Cng1|AFR92136.1<br>Cng1|AFR93600.1<br>Cng1|AFR93601.1<br>Cng1|AFR94598.1<br>Cng1|AFR95805.1<br>Cng1|AFR96830.1<br>Cng1|AFR97280.1<br>Cng1|AFR97665.1<br>Cng1|AFR97667.1<br>Cng1|AFR98190.2<br>Cng1|AFR98218.1	102/355	28,73%
RFLQPGDLVVLKS	predicted protein	150412547	EDN07934	02436	Hc01|EEH10906.1<br>Hc02|EGC45872.1<br>Hc03|EER42938.1<br>Hc04|EDN07934.1	Afl1|EED50243.1<br>Afu1|EAL84739.1<br>An01|OWW38395.1<br>At01|EAU38528.1<br>Tm01|XP_002153137.1<br>Ec01|KKZ67748.1<br>Ep01|KLJ07866.1<br>Bp01|OJD23091.1<br>Pl01|XP_015700471.1<br>Pb02|EEH48260.1<br>Pb01|EEH18807.2<br>Cp01|KMM69399.1<br>Ci01|KMP06183.1<br>Bd01|EGE85054.2<br>Fp01|CZR35185.1<br>Fo02|PCD45241.1<br>Fo01|KNA93747.1<br>Pj01|KTW31939.1<br>Cg01|ADV21634.1<br>Cg03|KIR59667.1<br>Cnn1|AAW46489.1<br>Cnn2|EAL18961.1<br>Cg02|KIS02342.1<br>Cng1|AFR94428.1<br>Ct01|EER34988.1<br>Sc01|EFI97097.1<br>Ca01|AOW27843.1<br>Ca02|EEQ47366.1<br>Cpa1|CCE42121.1	4/33	12,12%
KLSTLVGALATRN	predicted protein	150413459	EDN08842	Singletons - Hc04				

petide sequence	Protein Description	NCBI accesion	ID_proteom	cluster_groups	Hc_proteins cluster	Proteins_cluster	# Proteins_cluster (Hc/total)	%_Hc-proteins
RVAEEMRCKV	pre-mRNA splicing factor	240274117	EER37635	00082	Hc01|EEH06053.1<br>Hc01|EEH06733.1<br>Hc01|EEH07778.1<br>Hc01|EEH06598.1<br>Hc01|EEH11080.1<br>Hc02|EGC41887.1<br>Hc02|EGC46053.1<br>Hc02|EGC47401.1<br>Hc02|EGC47542.1<br>Hc02|EGC48810.1<br>Hc03|EER36487.1<br>Hc03|EER37635.1<br>Hc03|EER38394.1<br>Hc04|EDN02977.1<br>Hc04|EDN03097.1<br>Hc04|EDN08092.1<br>Hc04|EDN04099.1<br>Hc04|EDN10965.1<br>Hc03|EER43120.1<br>Hc04|EDN07166.1<br>Hc01|EEH11437.1<br>Hc03|EER39253.1<br>Hc02|EGC46423.1	Afl1|EED45712.1<br>Afl1|EED50529.1<br>Afl1|EED53015.1<br>Afl1|EED55580.1<br>Afl1|EED58151.1<br>Afu1|EAL88094.1<br>Afu1|EAL91450.1<br>Afu1|EAL91525.1<br>Afu1|EAL93068.1<br>An01|OWW27749.1<br>An01|OWW33448.1<br>An01|OWW35174.1<br>At01|EAU30680.1<br>At01|EAU31902.1<br>At01|EAU32280.1<br>At01|EAU35631.1<br>At01|EAU37071.1<br>Bd01|EGE79057.1<br>Bd01|EGE81686.2<br>Bd01|EGE81812.1<br>Bd01|EGE84822.1<br>Bd01|EGE79672.1<br>Bp01|OJD20646.1<br>Bp01|OJD22498.1<br>Bp01|OJD27623.1<br>Bp01|OJD20736.1<br>Bp01|OJD26358.1<br>Ca01|AOW28198.1<br>Ca01|AOW28443.1<br>Ca01|AOW29941.1<br>Ca01|AOW31003.1<br>Ca02|EEQ43363.1<br>Ca02|EEQ46548.1<br>Ca02|EEQ44225.1	23/188	12,23%
ACTLAAEPDR	2-isopropylmalate synthase	154282923	EDN05825	00398	Hc02|EGC44328.1<br>Hc01|EEH09857.1<br>Hc04|EDN05825.1<br>Hc03|EER38197.1<br>Hc03|EER38196.1<br>Hc02|EGC44327.1<br>Hc01|EEH09856.1	An01|OWW31231.1<br>An01|OWW32599.1<br>Ca01|AOW25719.1<br>Ca01|AOW27953.1<br>Ca02|EEQ43111.1<br>Ca02|EEQ47471.1<br>Cpa1|CCE40925.1<br>Cpa1|CCE42116.1<br>Ct01|EER31509.1<br>Ct01|EER35146.1<br>Tm01|XP_002146869.1<br>Tm01|XP_002146870.1<br>Afl1|EED57768.1<br>At01|EAU39494.1<br>Afu1|EAL90831.1<br>Ec01|KKZ64136.1<br>Ep01|KLJ05982.1<br>Pl01|XP_015699812.1<br>Pb01|EEH15817.2<br>Bp01|OJD22984.1<br>Pb02|EEH50755.2<br>Ci01|KMP07494.1<br>Cp01|KMM72626.1<br>Bd01|EGE80903.2<br>Fp01|CZR40239.1<br>Fo01|KNA97765.1<br>Fo02|PCD38290.1<br>Sc01|EFI99992.1<br>Cnn1|AAW45027.1<br>Cnn2|EAL17631.1<br>Cg03|KIR58021.1<br>Cg01|ADV25204.1<br>Cg02|KIR96516.1<br>[illegible]	7/71	9,86%
KAVLASEPRT	glycerol:H+ symporter	239610650				Blastomyces dermatitidis		

Supplemental Table S2. Comparative analysis between urine-peptides database from *Histoplasma* -immunoassay-positive patients and OrthoMCL analysis. For each peptide obtained from the urine-proteome associated with Histoplasma-antigenuria was described the NCBI_ accession, and the cluster groups with the orthologous proteins obtained by OrthoMCL analysis.
